# Prenylated acylphloroglucinols from the fruits of *Hypericum patulum*

**DOI:** 10.1007/s13659-026-00629-9

**Published:** 2026-04-23

**Authors:** Yu-Feng Qiu, Yi Zhou, Cheng Chen, Juan Huang, Xing-Wei Yang

**Affiliations:** 1 School of Pharmaceutical Sciences (Shenzhen), Sun Yat-Sen University, Shenzhen, 518107 People’s Republic of China; 2 State Key Laboratory of Cardiovascular Disease, Fuwai Shenzhen Hospital, Chinese Academy of Medical Sciences, Shenzhen, 518057 China; 3 Department of Pharmacology, Shanghai Medicilon Inc., Shanghai, China

**Keywords:** Prenylated acylphloroglucinols, *Hypericum patulum*, Structural determination, Cytotoxicity

## Abstract

Twenty-eight prenylated acylphloroglucinols, including the new hypulatones C–F (**1**–**4**), have been isolated from the fruits of *Hypericum patulum* and structurally characterized. Compound **1** represents a rare meroterpenoid formed through the addition of a prenylated acylphloroglucinol unit and a sesquiterpenoid moiety. Spirocyclic polycyclic polyprenylated acylphloroglucinol **2** contains six chiral centers, and its relative configuration was established based on ^1^H–^1^H coupling constants, conformational analysis, and NOE correlations. The cytotoxic activities of all isolates against two human carcinoma cell lines (Huh-7 and Panc-1) were evaluated using the CCK-8 assay. Bioassay results indicated that compounds **6**, **8**, and **15** exhibited moderate antiproliferative activity.

**Graphical Abstract:**

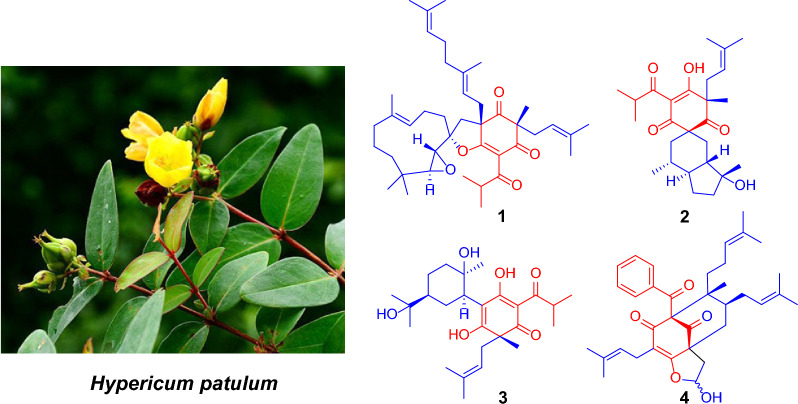

**Supplementary Information:**

The online version contains supplementary material available at 10.1007/s13659-026-00629-9.

## Introduction

**Fig. 1:**
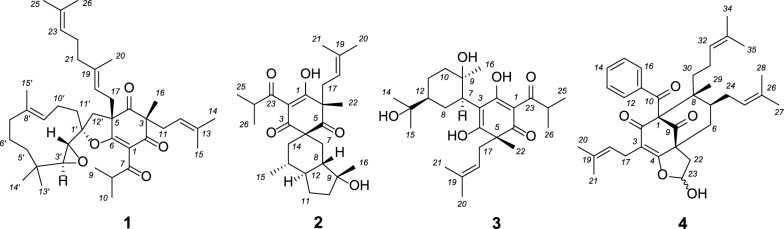
The structures of compounds **1**–**4**

**Fig. 2:**
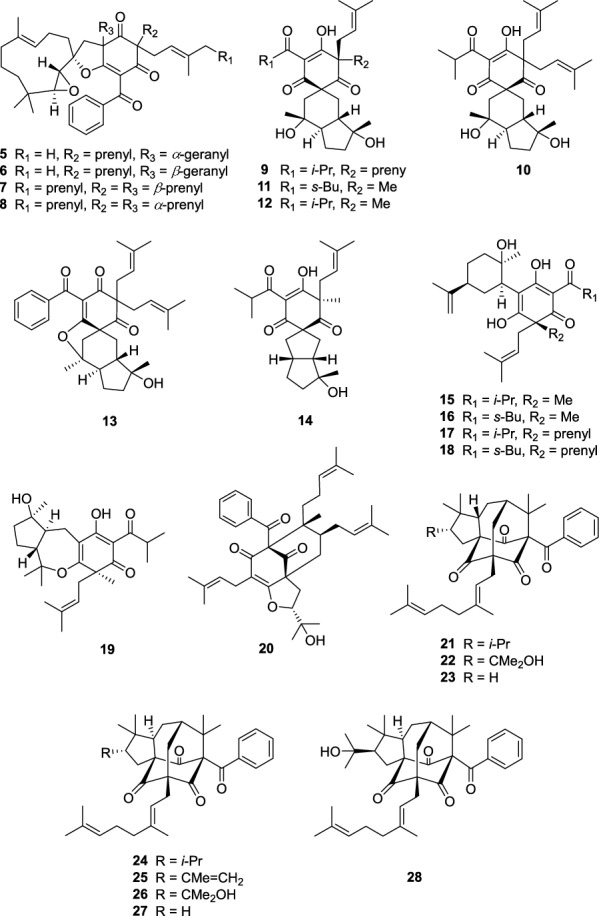
The structures of compounds **5**–**28**

Polyprenylated acylphloroglucinols (PAPs) are a class of structurally fascinating hybrid natural products that exhibit diverse bioactivities, such as tumor inhibitive, antimicrobial, HIV preventative, antioxidant, and antidepressant [1, 2]. Plants of the genus *Hypericum* (Hypericaceae) are known to be a rich source of PAPs. *Hypericum patulum*, commonly known as "Jinsimei", is a *Hypericum* species primarily distributed in southwestern China, notably in Yunnan, Guizhou, and Sichuan provinces [3]. It is a traditional Chinese medicine commonly used for the treatment of hepatitis, bacterial diseases, and epistaxis [4]. Due to the structural diversity of its chemical constituents and their potential pharmacological effects, this plant has attracted widespread scientific interest [5–17]. As we have a longstanding interest in the investigation of structures and bioactivities of PAPs [1, 18–30], we selected the fruit of *H. patulum* for further investigation. Four undescribed PAPs, hypulatones C–F (**1**–**4**), were isolated together with twenty-four known analogues (**5**–**28**, Figs. 1 and 2). Compound **1** represents a rare meroterpenoid formed through the addition of a prenylated acylphloroglucinol unit and a sesquiterpenoid moiety. To date, only eight enantiomeric pairs of this type of meroterpenoids have been reported [14, 31]. Herein are described the isolation, structure determination, and inhibitory activities against two human carcinoma cells (Huh-7 and Panc-1) of all the compounds isolated. This study provides an effective method for configurational assignments of spirocyclic polycyclic polyprenylated acylphloroglucinols (PPAPs) that bear six chiral centers.

## Results and discussion

**Fig. 3:**
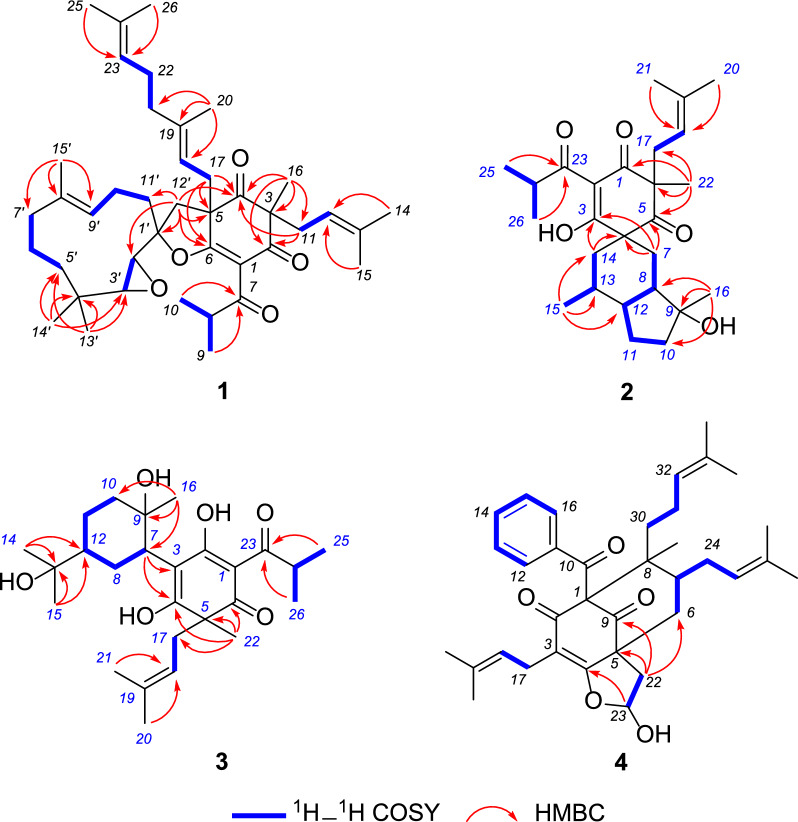
^1^H–^1^H COSY and HMBC correlations of **1**–**4**

Hypulatone C (**1**) was obtained as yellow gum. Its molecular formula C_41_H_60_O_5_ with 12 degrees of unsaturation was established by analysis of ^13^C NMR (Table 1) and HRESIMS data (*m/z* 671.4082, [M + K]^+^, cacld for 671.4072). The FTIR spectrum displayed absorption bands due to carbonyl (1719 and 1699 cm^−1^) functionalities. The ^1^H NMR spectrum (Table 1) exhibited the signals of an isopropyl (*δ*_H_ 3.07, sept; 1.16, d; 1.09, d; *J* = 6.9 Hz), four olefinic protons (*δ*_H_ 4.51–5.01), and nine singlet methyls (*δ*_H_ 0.64–1.67). Analysis of its ^13^C and DEPT-NMR data revealed a total of 41 carbon resonances, including a shielded *sp*^2^ carbon at *δ*_C_ 115.3 (C-1) and three deshielded carbons at *δ*_C_ 178.7 (C-6), 199.3 (C-2), and 203.2 (C-7), which were indicative of the presence of an enol-*β*-triketo system. The above signals, in combination with a nonconjugated ketone at *δ*_C_ 207.4 (C-4) and two quaternary carbons at *δ*_C_ 57.4 (C-3) and 62.6 (C-5), suggested that compound **1** should possess a dearomatized acylphloroglucinol core. This assumption was further confirmed by the HMBC correlations from a singlet methyl at *δ*_H_ 1.31 (Me-16) to C-2, C-3, and C-4, and from *δ*_H_ 3.29 and 2.28 (H_2_-17) to C-4, and C-5, and C-6. An isoprenyl group (*δ*_C_ 39.5, C-11; 118.6, C-12; 135.3, C-13; 26.0, C-14; 17.4, C-15) linked to C-3 was deduced by the correlation of *δ*_H_ 4.82 (H-12)/*δ*_H_ 2.67 and 2.31 (H_2_-11) in the ^1^H–^1^H COSY spectrum, together with the correlations of both *δ*_H_ 1.58 (Me-14) and 1.53 (Me-15) with C-11 and C-12, of H_2_-11 with C-2, C-3, and C-4 in the HMBC spectrum. Moreover, C-17 was proved to be the head of a geranyl group (*δ*_C_ 40.3, C-17; 116.6, C-18; 141.4, C-19; 17.6, C-20; 39.9, C-21; 26.3, C-22; 123.9, C-23; 131.5, C-24; 25.7, C-25; 17.7, C-26) by the HMBC correlations from *δ*_H_ 1.62 (Me-20) to C-18, C-19, and C-21, from both *δ*_H_ 1.67 (Me-25) and 1.59 (Me-26) to C-23 and C-24, coupled with the proton spin systems of H_2_-17/*δ*_H_ 4.74 (H-18) and *δ*_H_ 1.97 (H_2_-21)/*δ*_H_ 2.03 (H_2_-22)/*δ*_H_ 5.02 (H-23) in the ^1^H–^1^H COSY spectrum. The HMBC correlations of both doublet methyls at *δ*_H_ 1.16 and 1.09 with C-7 assigned the location of the isopropyl group (*δ*_C_ 39.8, C-8; 18.5, C-9; 18.1, C-10) (Fig. 3).

**Table 1:** ^13^C (150 MHz) and ^1^H NMR (600 MHz) data of compound **1** in CDCl_3_

No	*δ*_C_, type	*δ*_H_ mult. (*J* in Hz)	No	*δ*_C_, type	*δ*_H_ mult. (*J* in Hz)
1	115.3, C		23	123.9, CH	5.02, t (6.3)
2	199.3, C		24	131.5, C	
3	57.4, C		25	25.7, CH_3_	1.67, s
4	207.4, C		26	17.7, CH_3_	1.59, s
5	62.6, C		1′	92.4, C	
6	178.7, C		2′	59.4, CH	2.63, d (2.0)
7	203.2, C		3′	64.2, CH	2.85, d (2.0)
8	39.8, CH	3.07, sept (6.9)	4′	33.8, C	
9	18.5, CH_3_	1.09, d (6.9)	5′	39.3, CH_2_	1.57, overlap
10	18.1, CH_3_	1.16, d (6.9)			1.24, m
11	39.5, CH_2_	2.67, dd (13.8, 7.8)	6′	20.2, CH_2_	1.57, overlap
		2.31, dd (13.8, 7.8)			1.41, m
12	118.6, CH	4.82, t (7.8)	7′	39.5, CH_2_	2.02, overlap
13	135.3, C				1.76, t (12.6)
14	26.0, CH_3_	1.58, s	8′	135.5, C	
15	17.4, CH_3_	1.53, s	9′	123.6, CH	5.13, dd (10.2, 4.0)
16	21.8, CH_3_	1.31, s	10′	23.3, CH_2_	2.41, m
17	40.3, CH_2_	3.29, dd (15.0, 9.3)			1.95, overlap
		2.28, dd (15.0, 4.0)	11′	40.3, CH_2_	1.97, overlap
18	116.6, CH	4.74, dd (9.3, 4.0)			1.71, m
19	141.4, C		12′	41.0, CH_2_	2.31, s
20	17.6, CH_3_	1.62, s	13′	25.3, CH_3_	1.00, s
21	39.9, CH_2_	1.97, m	14′	22.4, CH_3_	0.64, s
22	26.3, CH_2_	2.03, m	15′	16.3, CH_3_	1.64, s

Besides the aforementioned 26 carbon signals in the ^13^C and DEPT NMR spectra of **1**, the remaining 15 resonances assignable to three nonprotonated carbons (*δ*_C_ 135.5, C-8′; 92.4, C-1′; and 33.8, C-4′), three methines (*δ*_C_ 123.6, C-9′; 64.2, C-3′; and 59.4, C-2′), six methylenes, and three methyls indicated a humulane-type sesquiterpenoid moiety. This deduction was further confirmed by the correlations of 2.63 (H-2′)/*δ*_H_ 2.85 (H-3′), and *δ*_H_ 1.24 (H-5′)/*δ*_H_ 1.41 (H-6′)/*δ*_H_ 1.76 (H-7′), and *δ*_H_ 5.13 (H-9′)/*δ*_H_ 2.41 (H-10′)/*δ*_H_ 1.71 (H-11′) in the ^1^H–^1^H COSY spectrum, along with the HMBC correlations from *δ*_H_ 2.31 (H-12′) to *δ*_C_ 92.4 (C-1′), 59.4 (C-2′), and 40.3 (C-11′), from both singlet methyls at *δ*_H_ 0.64 (Me-14′) and 1.00 (Me-13′) to *δ*_C_ 64.2 (C-3′), 33.8 (C-4′), and 39.3 (C-5′), and from singlet methyl at *δ*_H_ 1.64 (H-15′) to *δ*_C_ 39.5 (C-7′), 135.5 (C-8′), and 123.6 (C-9′) (Fig. 3).

The linkage of C-5/C-12′ was deduced by the HMBC correlations from H_2_-12′ to C-4, C-5, and C-6, which combined the acylphloroglucinol and sesquiterpenoid moieties. The formation of the 2′,3′-epoxide and deduced furan ring were indicated by the indices of hydrogen deficiency along with the special chemical shifts of C-1′ (*δ*_C_ 92.4), C-2′ (*δ*_C_ 59.4), C-3′ (*δ*_C_ 64.2), and C-6 (*δ*_C_ 178.7). So far, the planar structure of **1** was elucidated as shown (Fig. 1).

**Fig. 4:**
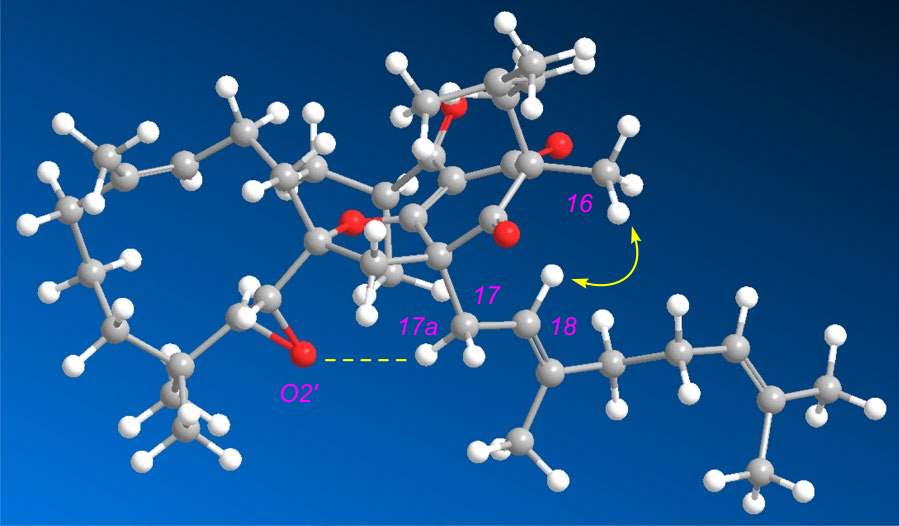
Configuration optimized molecular model of **1**. yellow arrows, key NOE correlation; yellow dashed line, close O2′/H-17a contact

Comparison of the structure of **1** with that of hyperkouytin C (**6**) [31] indicated that the benzoyl and prenyl groups in **6** were replaced by an isobutyryl and a methyl in **1**, respectively. The ^13^C chemical shifts of carbons around C-1′, C-2′ C-3′, and C-5 chiral centers are very close to those of **6**, which suggests that the relative configurations of C-1′, C-2′ C-3′, and C-5 are identical to those of **6**. Furthermore, the chemical shift of H-17a (*δ*_H_ 3.29) was 1.01 ppm downfield of H-17b (*δ*_H_ 2.28), indicating that compound **1** had a close O2′/H-17a contact and the geranyl group was located at the same side of C-2′. This deduction was confirmed by a computationally optimized model (Fig. 4) and the crystallographic data of hypulatone B and hyperkouytins A and B [14, 31]. These evidences further confirmed the relative configurations four chiral carbons mentioned above. Finally, the NOESY correlation between Me-16 and H-18 established the configuration of C-3, thus assigning the relative configuration of **1** as 3S*, 5S*, 1'R*, 2'R*, 3'S* (Fig. 4). Considering that all the meroterpenoids of this type were reported in enantiomeric pairs [14, 31], the optical rotation of **1** ([*α*]_D_ =  + 19) suggested it might be scalemic mixtures. Nevertheless, the lack of sufficient sample quantities precluded the further chiral separation.

**Fig. 5:**
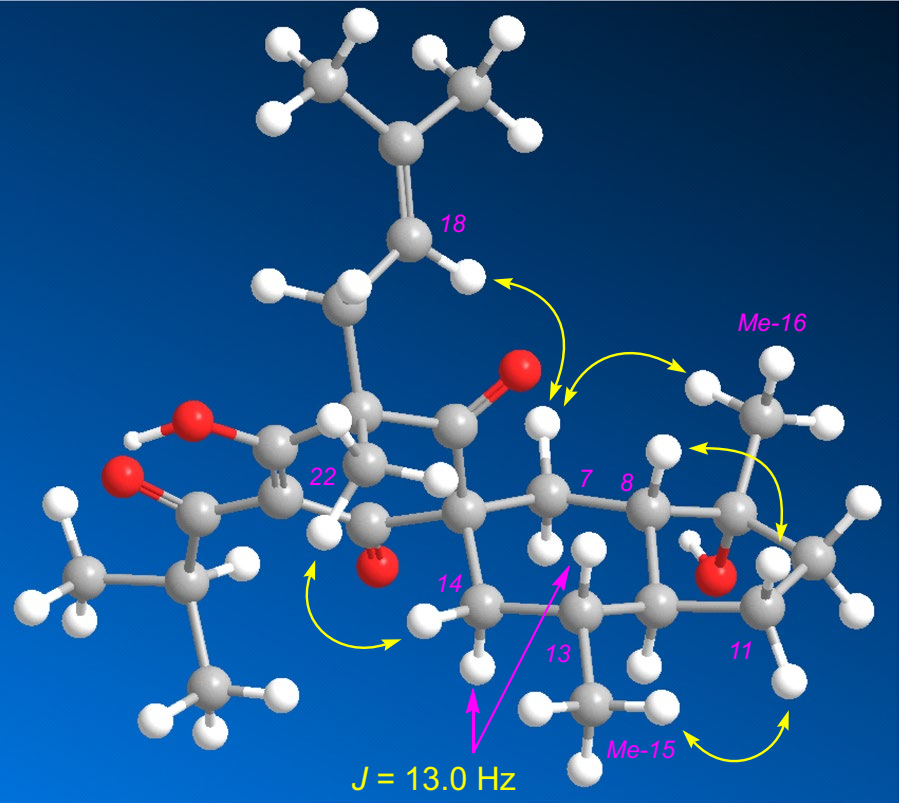
Configuration optimized molecular model of **2**. Pink arrows, coupling constants; yellow arrows, NOE correlations

Hypulatone D (**2**) was assigned the molecular formula C_26_H_38_O_5_ by analysis of its ^13^C NMR (Table 2) and HREIMS data (*m/z* 429.2643 [M − H]^−^). Comparing the ^1^H and ^13^C NMR data of **2** to those of hyperpatulone E [15] indicates that they are structurally similar. The *sec*-butyl group in hyperpatulone E is replaced by an isopropyl (*δ*_H_ 1.21, d; 1.13, d; 3.51, sept.; *J* = 6.8 Hz) in **2**, as evidenced by the HMBC correlations from Me-25 (*δ*_H_ 1.21) and Me-26 (*δ*_H_ 1.13) to *δ*_C_ 206.7 (C-23). It is worth noting that one could barely determine the relative configuration of spirocyclic PPAPs characterized by six chiral centers, like **2**, unless one used a combination of ^1^H–^1^H coupling constants, conformational analysis, and NOE correlations. Firstly, in the ^1^H spectrum of **2** measured in CDCl_3_ (Table S1), the ^3^*J* coupling constant of H-14b (*δ*_H_ 1.23, t, *J* = 13.0 Hz) was 13.0 Hz. So, the corresponding six-membered ring adopted chair conformations (Fig. 5), and H-14a (*δ*_H_ 2.00, brd, *J* = 13.0 Hz) and Me-15 were equatorial while H-14b and H-13 (*δ*_H_ 1.25) were axial. Secondly, the NOE contacts of Me-15 with H-11_eq_ (*δ*_H_ 1.86) and of H-11_ax_ (*δ*_H_ 1.11) with H-8 (*δ*_H_ 1.57) indicated the trans configuration of the octahydro-indene moiety, as well as the relationship between H-12 and H-13. Thirdly, due to the constraints of the spirocyclic framework, the phloroglucinol ring is perpendicular to the octahydro-indene moiety, which itself lies nearly coplanar with the plane formed by the two C-6 substituents. The NOE correlations of H-14a/Me-22 (*δ*_H_ 1.43), H-18 (*δ*_H_ 4.70)/H-7b (*δ*_H_ 1.79), and H-7b/Me-16 (*δ*_H_ 1.28) measured in CD_3_OD defined the relative configurations of C-6 and C-9. Thus, the structure of **2** was determined as shown and named as hypulatone D (Fig. 1).

**Table 2:** ^13^C (150 MHz) and ^1^H NMR (600 MHz) data of compounds **2**–**4**

No	**2** (CD_3_OD)		**3** (CD_3_OD)		**4** (CDCl_3_)	
	*δ*_C_, type	*δ*_H_ mult. (*J* in Hz)	*δ*_C_, type	*δ*_H_ mult. (*J* in Hz)	*δ*_C_, type	*δ*_H_ mult. (*J* in Hz)
1	198.5, C		105.6, C		79.7, C	
2	112.9, C		191.1, C		193.6, C	
3	198.8, C		111.3, C		116.7, C	
4	66.4, C		179.3, C		171.8, C	
5	210.0, C		54.8, C		58.4, C	
6	57.5, C		198.9, C		40.2, CH_2_	2.90, dd (14.0, 6.0)
						1.93, d (14.0)
7	27.1, CH_2_	1.86, t (13.0)	42.8, CH	3.18, dd (12.4, 3.2)	43.9, CH	1.84, overlap
		1.79, m				
8	51.3, CH	1.54, m	41.1, CH_2_	1.83, m	49.1, C	
				1.55, m		
9	79.8, C		73.2, C		204.7, C	
10	41.2, CH_2_	1.79, overlap	28.0, CH_2_	1.78, t (12.5)	194.0, C	
		1.71, m		1.43, m		
11	27.9, CH_2_	1.84, m	23.5, CH_2_	1.67, m	137.0, C	
		1.09, m		1.61, m		
12	49.9, CH	1.23, overlap	50.8, CH	1.48, m	128.1, CH	7.43, d (7.7)
13	36.4, CH	1.22, overlap	73.2, C		127.9, CH	7.22, t (7.8)
14	45.0, CH_2_	2.06, brd (10.1)	27.2, CH_3_	1.15, s	132.0, CH	7.38, t (7.7)
		1.21, overlap				
15	20.3, CH_3_	0.87, d (5.0)	26.8, CH_3_	1.14, s	127.9, CH	7.22, t (7.8)
16	26.7, CH_3_	1.28, s	28.0, CH_3_	1.16, s	128.1, CH	7.43, d (7.7)
17	40.3, CH_2_	2.64, dd (13.3, 8.2)	40.1, CH_2_	2.63, dd (14.0, 7.3)	22.4, CH_2_	3.11, m
		2.45, dd (13.3, 8.2)		2.58, dd (14.0, 6.4)		3.04, dd (14.1, 7.2)
18	118.9, CH	4.70, t (8.2)	120.5, CH	4.84, overlap	120.2, CH	5.09, overlap
19	138.1, C		135.4, C		132.9, C	
20	26.1, CH_3_	1.55, s	26.2, CH_3_	1.54, s	25.8, CH_3_	1.62, s
21	17.9, CH_3_	1.45, s	18.1, CH_3_	1.58, s	17.7, CH_3_	1.65, s
22	23.0, CH_3_	1.43, s	24.8, CH_3_	1.32, s	36.4, CH_2_	2.59, m
						1.71, overlap
23	206.7, C		208.5, C		102.4, CH	6.05, d (5.6)
					OH	3.62, brs
24	35.6, CH	3.51, sept (6.8)	36.8, CH	3.98, sept (6.8)	25.5, CH_2_	2.28, m
						1.99, m
25	20.1, CH_3_	1.21, d (6.8)	19.5, CH_3_	1.11, d (6.8)	124.7, CH	5.09, overlap
26	19.3, CH_3_	1.13, d (6.8)	19.3, CH_3_	1.10, d (6.8)	131.1, C	
27					25.8, CH_3_	1.63, s
28					18.0 CH_3_	1.65, s
29					13.7, CH3	1.19, s
30					36.4, CH_2_	2.21, overlap
						1.41, td (14.0, 5.0)
31					27.1, CH_2_	2.23, overlap
						1.84, overlap
32					122.2, CH	5.02, t (7.1)
33					133.4, C	
34					25.5, CH_3_	1.59, s
35					17.8, CH_3_	1.71, s

The molecular formula of hypulatone E (**3**) was determined as C_26_H_40_O_6_ by analysis of its ^13^C NMR (Table 2) and HRESIMS data (*m/z* 447.2745 [M–H]^−^). The ^1^H and ^13^C NMR data of **3** resembled those of hyperhenone E (**15**) [32]. Instead of two olefinic carbons in hyperhenone E, an oxygen-bearing quaternary carbon at *δ*_C_ 73.2 (C-13) and a methyl at *δ*_C_ 27.2 (Me-14) appeared in **3**, suggesting that **3** could be derived from hyperhenone E by adding water across the *Δ*^13,14^ double bond of the latter. This suggestion was further supported by the correlations of both Me-14 (*δ*_H_ 1.15) and Me-15 (*δ*_H_ 1.14) with C-13 and C-12 (*δ*_C_ 50.8). The NOE contacts of H-7 (3.18) with H-12 (1.48) and Me-16 (1.16) indicated that the relative configurations of C-7, C-9, and C-12 were identical to those of hyperhenone E. Furthermore, the well matched ECD curves of **3** and hyperhenone E (**15**) suggested that their absolute configuration of C-5 was identical [32–34]. Considering that compounds **3** and **15** were co-isolated and the absolute configuration of **15** was determined by X-ray diffraction data [33], the absolute configuration of **3** could be defined as *5R*, 7*R*, 9*R*, 12*S* (Fig. 1).

The molecular formula of hypulatone F (**4**) was determined to be C_35_H_44_O_5_ from its HRESIMS and ^13^C NMR data (Table 2). On the basis of analysis of its 1D and 2D NMR data, compound **4** was assigned to possess the same backbone as hypseudohenrin F [35]. The structural novelty of **4** involved the presence of a hemiacetal hydroxyl (*δ*_H_ 3.62, OH-23) rather than a methoxy group, which was confirmed by the ^1^H–^1^H COSY correlations of H-22a (*δ*_H_ 2.59) with H-23 (*δ*_H_ 6.05), in combination with the HMBC correlations from H-22a to C-5 (*δ*_C_ 58.4) and C-6 (*δ*_C_ 40.2) and C-9 (*δ*_C_ 204.7), and from H-23 to C-4 (*δ*_C_ 171.8) (Fig. 3). The 2D NMR data showed that the other structural features of **4** were identical to those of hypseudohenrin F.

Twenty-four known compounds were identified as hypulatone A (**5**) [14], ( +)-hyperkouytin C (**6**) [31], hypulatone B (**7**) [14], ( −)-hyperkouytin D (**8**) [31], tomoeone A (**9**) [36], tomoeone B (**10**) [36], chipericumin D (**11**) [37], chipericumin E (**12**) [38], hypercohone G (**13**) [39], spirohypatone A (**14**) [13], hyperhenone E (**15**) [32], bellumone I (**16**) [40], hyphenrone J (**17**) [23], hyphenrone K (**18**) [23], hyphenol J (**19**) [34], uralione E (**20**) [41], hookerione K (**21**) [42], attenuatumione D (**22**) [43], sampsonione H (**23**) [44], hypersampsone D (**24**) [45], sampsonione D (**25**) [44], sampsonione C (**26**) [44], hypersampsone I (**27**) [46], and hypersampsonone G (**28**) [47], by comparison of their spectroscopic and physical data with those of related literature (Fig. 2).

All the isolates (compounds **1**–**28**) were tested for their cytotoxic activities on Huh-7 and Panc-1 cell lines by CCK-8 assay. Sorafenib and paclitaxel were used as the positive control. As shown in Table 3, compounds **6**, **8**, and **15** showed moderate inhibitory activity against two human cancer cell lines with IC_50_ values in the range of 9.7–19.2 µM.

**Table 3:** Cytotoxicity of compounds **1**–**28** on two cancer cell lines with IC_50_ values (μM)

Compound	Huh-7	Panc-1	Compound	Huh-7	Panc-1
**1**	35.9 ± 3.3	48.4 ± 6.9	**16**	42.1 ± 2.8	45.4 ± 4.9
**2**	40.2 ± 1.1	> 50	**17**	23.0 ± 2.6	18.6 ± 1.2
**3**	> 50	41.4 ± 2.1	**18**	34.6 ± 0.4	23.4 ± 1.3
**4**	32.3 ± 3.7	35.5 ± 1.6	**19**	24.9 ± 2.5	> 50
**5**	17.3 ± 0.7	20.7 ± 1.8	**20**	17.5 ± 0.5	22.8 ± 2.7
**6**	19.2 ± 1.0	12.6 ± 0.8	**21**	36.8 ± 3.6	37.3 ± 2.5
**7**	21.8 ± 1.0	17.4 ± 0.4	**22**	10.0 ± 0.4	> 50
**8**	10.4 ± 0.2	15.5 ± 0.8	**23**	> 50	> 50
**9**	36.7 ± 0.7	> 50	**24**	> 50	> 50
**10**	43.0 ± 3.0	> 50	**25**	41.9 ± 2.3	48.5 ± 3.4
**11**	29.6 ± 0.9	> 50	**26**	27.3 ± 0.6	21.7 ± 1.6
**12**	> 50	> 50	**27**	30.8 ± 0.7	42.0 ± 3.8
**13**	27.2 ± 0.8	> 50	**28**	44.0 ± 2.5	16.3 ± 1.0
**14**	20.3 ± 0.5	20.4 ± 0.6	Sorafenib	7.4 ± 0.4	8.3 ± 1.3
**15**	9.7 ± 1.0	11.5 ± 0.5	Paclitaxel	8.1 ± 0.7	3.2 ± 1.3

In summary, four previously undescribed PAPs, hypulatones C–F (**1**–**4**), together with twenty-four known analogues, were isolated from the fruit of *Hypericum patulum* and structurally characterized*.* Compounds **6**, **8**, and **15** showed moderate inhibitory activity against two human cancer cell lines with IC_50_ values in the range of 9.7–19.2 µM. Our findings enriched the structural diversity of natural PAPs, and also provided a useful method for configurational assignments of spirocyclic PPAPs that bear six chiral centers.

## Experimental procedures

### General experimental procedures

Optical rotations were measured on a Jasco P-1020 polarimeter. UV spectra were recorded on a Shimadzu UV-2401PC spectrometer. IR spectra were recorded on a Bruker FT-IR Tensor-27 infrared spectrophotometer with KBr disks. 1D and 2D NMR spectra were recorded on a Bruker DRX-600 spectrometer using TMS as an internal standard. Unless otherwise specified, chemical shifts (*δ*) are expressed in ppm with reference to the solvent signals. ESIMS and HREIMS data were acquired on Waters Xevo TQS and Waters AutoSpec Premier P776 mass spectrometers, respectively. Semi-preparative HPLC was performed on an Agilent 1100 HPLC with a Zorbarx SB-C_18_ (9.4 × 250 mm) column. Silica gel (200–300 mesh, Qingdao Marine Chemical Co., Ltd., Qingdao, People’s Republic of China) were used for column chromatography. Fractions were monitored by TLC (GF 254, Qingdao Marine Chemical Co., Ltd.), and spots were visualized by heating silica gel plates immersed in H_2_SO_4_ in EtOH.

### Plant materials

The fruits of *Hypericum patulum* were collected in Xishan of Kunming County, Yunan Province, People’s Republic of China, in July 2020. The plant was identified by Dr. L. Zhang, and a voucher specimen (KIB 20200701) has been deposited at the Kunming Institute of Botany.

### Extraction and isolation

The dried fruits of *Hypericum patulum* (3.12 kg) were powdered and percolated with MeOH (3 × 10 L) at room temperature for 24 h to yield an extract (680 g) after evaporation in vacuo. The residue was suspended in H_2_O and partitioned with EtOAc and H_2_O to yield the EtOAc fraction (350 g). This fraction was subjected to column chromatography over silica gel eluted with a petroleum ether–EtOAc in gradient (19:1, 9:1, 8:2, 7:3, 1:1, and 0:1) to obtain 10 fractions (Fr. A–J).

Fr. A (18.6 g) was fractionated by MCI gel column chromatography (MeOH–H_2_O, 70:30–100:0) to provide five subfractions (Fr. A1–A5). Fr. A3 (3.1 g) was fractionated by silica column chromatography using petroleum ether–EtOAc (100:1–0:1) as eluents to provide four subfractions (Fr. A3.1–A3.4). Fr. A3.2 (715 mg) was then purified by semipreparative HPLC (MeOH–H_2_O, 92:8) to produce compounds **23** (9.1 mg) and **27** (30.8 mg). Fr. A4 (1.9 g) was then fractionated by silica gel column chromatography using petroleum ether–EtOAc (50:1–0:1) as eluents to provide four subfractions (Fr. A4.1–A4.4). Fr. A4.2 (540 mg) was then purified by semipreparative HPLC eluting with MeOH–H_2_O (95:5), in combination with preparative TLC, to afford compounds **1** (2.6 mg), **7** (4 mg), **21** (7 mg), **24** (9.9 mg) and **25** (6 mg). Fr. A4.3 (175 mg) was then fractionated by semipreparative HPLC (MeOH–H_2_O, 94:6) to produce compounds **5** (8 mg), **6** (1.3 mg), and **8** (15.7 mg). A portion of Fr. B (6.8 g) was fractionated by MCI gel column chromatography (MeOH–H_2_O, 60:40–100:0) to provide five subfractions (Fr. B1–B5). Fr. B5 (3.5 g) was chromatographed on a silica gel column, eluting with petroleum ether–EtOAc (20:1–0:1), to gather Fr. B5.1–B5.3. Compounds **16** (3.9 mg), **17** (6 mg), and **18** (4.1 mg) were obtained from Fr. B5.2 by semipreparative HPLC (MeOH–H_2_O, 95:5). Using semipreparative HPLC (MeCN–H_2_O, 85:15 and 70:30, respectively), Fr. B4 (639 mg) afforded compounds **10** (4.8 mg) and **15** (89.4 mg). Fr. C (38.5 g) was fractionated by using MCI gel column chromatography (MeOH–H_2_O, 40:60–100:0) to gather Fr. C1–C5. Fr. C5 was chromatographed on a silica gel column (petroleum ether–EtOAc, 20:1–0:1) to provide five subfractions (Fr. C5.1–C5.5). Using semipreparative HPLC (MeCN–H_2_O, 85:15) and preparative TLC (petroleum ether–EtOAc), compounds **11** (33 mg), **20** (5 mg), **28** (28.1 mg) from Fr. C5.4 (889.5 mg), and compounds **4** (12.3 mg), **9** (3.2 mg), **22** (18.3 mg), and **26** (28.3 mg) from Fr. C5.5 (48.9 mg) were isolated. Similarly, compounds **2** (3.1 mg), **3** (4.5 mg), **12** (13.2 mg), **13** (3.1 mg), **14** (6.3 mg), and **19** (3.9 mg) were obtained from Fr. E (60 g) by using silica gel column, semipreparative HPLC (MeCN − H_2_O, 70:30), and preparative TLC.

*Hypulatone C (****1****):* Yellow gum; $[\alpha]^{25}_{D}$ + 19 (*c* 0.1, MeOH); UV (MeOH) *λ*_max_ (log *ε*) 210 (4.28), 230 (4.31), 275 (4.28) nm; IR (KBr) *ν*_max_ 2964, 2930, 2872, 2857, 1719, 1699, 1625, 1456, 1383, 1250, 1184, 1095, 1030, 908, 803 cm^−1^; ^1^H and ^13^C NMR data, see Table 1. ESIMS *m/z* 671 [M + K]^+^; HRESIMS *m/z* 671.4082 (calcd for C_41_H_60_O_5_K, 671.4072).

*Hypulatone D (****2****):* Yellow gum; $[\alpha]^{25}_{D}$ − 12 (*c* 0.1, MeOH); UV (MeOH) *λ*_max_ (log *ε*) 240 (2.89), 283 (3.02) nm; CD (*c* 3 × 10^−4^, MeOH) *λ*_max_ nm (Δ*ε*) 201 (− 5.23), 221 (+ 10.95), 244 (+ 9.96), 272 (+ 9.50), 295 (− 1.82), 305 (+ 1.13), 334 (− 2.83); IR (KBr) *ν*_max_ 3317, 2945, 2831, 1668, 1533, 1448, 1279, 1022 cm^−1^; ^1^H and ^13^C NMR data, see Table 2. ESIMS *m/z* 429 [M − H]^−^; HRESIMS *m/z* 429.2643 (calcd for C_26_H_37_O_5_, 429.2641).

*Hypulatone E (****3****):* Light yellow gum; $[\alpha]^{25}_{D}$ + 112 (*c* 0.1, MeOH); UV (MeOH) *λ*_max_ (log *ε*) 228 (3.07), 238 (3.02), 279 (2.90), 327 (2.97) nm; CD (*c* 3 × 10^−4^, MeOH) *λ*_max_ nm (Δ*ε*) 199 (− 18.6), 228 (+ 4.52), 248 (+ 0.29), 270 (+ 7.74), 317 (+ 10.39), 350 (+ 5.35); IR (KBr) *ν*_max_ 3334, 3084, 1637, 1502, 1022 cm^−1^; ^1^H and ^13^C NMR data, see Table 2. ESIMS *m/z* 447 [M − H]^−^; HRESIMS *m/z* 447.2745 (calcd for C_26_H_39_O_6_, 447.2747).

*Hypulatone F (****4****):* Light yellow gum; $[\alpha]^{25}_{D}$ − 23 (*c* 0.1, MeOH); UV (MeOH) *λ*_max_ (log *ε*) 210 (4.25), 248 (4.30), 275 (4.18) nm; IR (KBr) *ν*_max_ 3437, 2967, 2925, 2859, 1728, 1695, 1627, 1448, 1340, 1317, 1227, 1190,1142, 1057,869, 799, 768, 689 cm^−1^; ^1^H and ^13^C NMR data, see Table 2. HRESIMS *m/z* 583.2826 (calcd for C_35_H_44_O_5_K, 583.2820).

### Cytotoxicity assay

Cells (Huh-7 and Panc-1) were seeded in 96-well plates at a density of 1 × 10^4^ cells per well, incubated for 24 h, and treated with the different concentrations of all isolated compounds (3.125, 6.25, 12.5, 25, and 50 μM) at 37 °C in 5% CO_2_ for another 24 h. A 10 μL amount of the Cell Counting Kit-8 (Biosharp, Shanghai, China) was added to the medium and incubated for 2 − 4 h; then the absorbance was read at a wavelength of 450 nm using a microplate reader (Shenzhen Sanli Technology Co. Ltd, Shenzhen, China). Sorafenib (G-CLONE, Beijing, China) and paclitaxel (G-CLONE, Beijing, China) were used as positive control [27]. The half-maximal inhibitory concentration (IC_50_) value was measured and calculated by GraphPad Prism 8 software.

## Supplementary Information

Supplementary material 1. ^1^H and ^13^C NMR data for compounds **2** and **3** in CDCl_3_ (Table S1), Original MS and NMR spectra of compounds **1**–**4** (Figs. S1–S44).

## Data Availability

All data generated or analyzed during this study are included in this published article and its supplementary information files.
