# Supplementary material for: Prenylated acylphloroglucinols from the fruits of Hypericum patulum
Source: Nat Prod Bioprospect. 2026 Apr 23;16(1):57. doi: 10.1007/s13659-026-00629-9 (PMC13103025; doi:10.1007/s13659-026-00629-9)
Supplement: Supplementary file 1 — Supplementary material 1. 1H and 13C NMR data for compounds 2 and 3 in CDCl3 (Table S1), Original MS and NMR spectra of compounds 1–4 (Figs. S1–S44). [file 13659_2026_629_MOESM1_ESM.pdf]

Prenylated acylphloroglucinols from the fruits of *Hypericum patulum*

Yu-Feng Qiu,[1†] Yi Zhou,[1†] Cheng Chen,[3] Juan Huang,[2,*] and Xing-Wei Yang[1,*]

[1] School of Pharmaceutical Sciences (Shenzhen), Sun Yat-sen University, Shenzhen 518107, People's Republic of China

[2] State Key Laboratory of Cardiovascular Disease, Fuwai Shenzhen Hospital, Chinese Academy of Medical Sciences, Shenzhen 518057, China

[3] Department of Pharmacology, Shanghai Medicilon Inc., Shanghai, China

[†] Yu-Feng Qiu and Yi Zhou have authors contributed equally

Supplementary Information

Table of Contents

Table S1. ^{1}H and ^{13}C NMR data for compounds **2** and **3** in $CDCl_3$ (Page S2)

The original MS and NMR spectra of compound **1** (Page S3–S7)

The original MS and NMR spectra of compound **2** (Page S8–S14)

The original ECD and NMR spectra of compound **3** (Page S15–S21)

The original MS and NMR spectra of compound **4** (Page S22–S26)

Table S1. ^{1}H (600 MHz) and ^{13}C NMR (150 MHz) data for compounds **2** and **3** in $CDCl_3$.

	**2**		**3**	
no.	δ_C, type	δ_H mult. (*J* in Hz)	δ_C, type	δ_H mult. (*J* in Hz)
1	198.3, C		105.8, C	
2	111.2, C		190.0, C	
3	197.3, C		109.9, C	
4	64.8, C		176.8, C	
5	208.7, C		53.4, C	
6	56.4, C		196.9, C	
7	25.9, CH_2	1.82, m	40.6, CH	3.27, dd (13.4, 3.4)
8	49.8, CH	1.57, m	40.4, CH_2	1.81, m
				1.70, m
9	79.3, C		74.6, C	
10	40.0, CH_2	1.83, m	26.4, CH_2	1.77, m
		1.72, m		1.51, overlap
11	26.7, CH_2	1.86, m	22.2, CH_2	1.75, m
		1.11, m		1.54, overlap
12	48.5, CH	1.24, m	48.9, CH	1.52, m
13	34.8, CH	1.25, overlap	72.6, C	
14	45.0, CH_2	2.00, brd (13.0)	27.4, CH_3	1.21, s
		1.23, t (13.0)		
15	20.0, CH_3	0.86, d (5.2)	27.3, CH_3	1.21, s
16	26.7, CH_3	1.34, s	27.7, CH_3	1.24, s
17	39.2, CH_2	2.67, dd (13.3, 7.8)	39.0, CH_2	2.63, dd (13.4, 8.0)
		2.45, dd (13.3, 7.8)		2.58, dd (13.4, 8.0)
18	117.5, CH	4.71, t (7.8)	119.4, CH	4.83, t (8.0)
19	137.1, C		134.1, C	
20	25.9, CH_3	1.56, s	25.9, CH_3	1.55, s
21	17.7, CH_3	1.46, s	18.0, CH_3	1.57, s
22	22.6, CH_3	1.45, s	24.0, CH_3	1.37, s
23	206.2, C		206.9, C	
24	34.3, CH	3.59, sept (6.8)	35.2, CH	4.03, sept (7.1)
25	19.7, CH_3	1.22, d (6.8)	19.0, CH_3	1.14, d (7.1)
26	19.0, CH_3	1.15, d (6.8)	19.0, CH_3	1.15, d (7.1)
4-OH				11.77, s

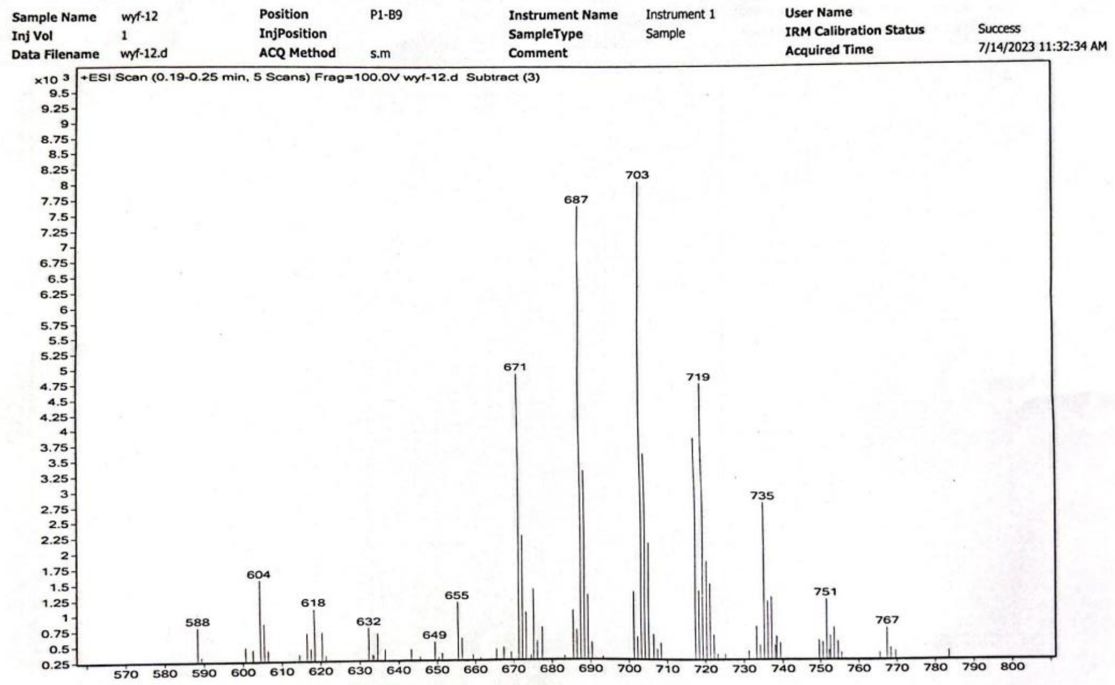

Figure S1. ESIMS spectrum of **1**.

Qualitative Analysis Report

**Data Filename**	wyf-12.d	**Sample Name**	wyf-12
**Sample Type**	Sample	**Position**	P1-B9
**Instrument Name**	Instrument 1	**User Name**	
**Acq Method**	s.m	**Acquired Time**	7/14/2023 1:21:09 PM
**IRM Calibration Status**	Success	**DA Method**	PCDL.m
**Comment**			

**Sample Group**		**Info.**
**Acquisition SW Version**	6200 series TOF/6500 series Q-TOF B.05.01 (B5125.2)	

User Spectra

Fragmentor Voltage	Collision Energy	Ionization Mode
175	0	ESI

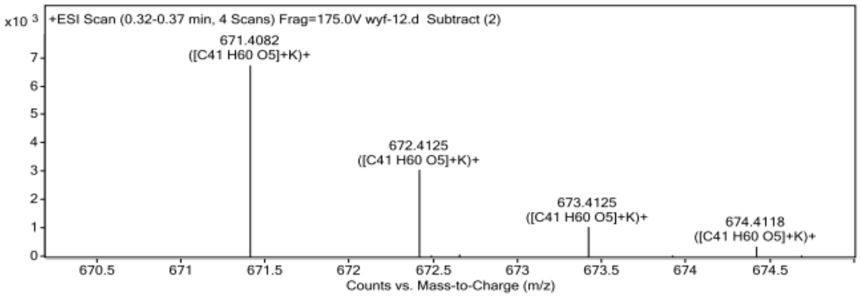

Peak List

m/z	z	Abund	Formula	Ion
156.845	1	4282.89		
158.8432	1	4527.83		
671.4082	1	6758.8	C41 H60 O5	(M+K)+
672.4125	1	3082.15	C41 H60 O5	(M+K)+
687.4038	1	4539.44		
688.4088	1	1863.68		
701.4045	1	1888.32		
703.3985	1	4546.45		
704.4026	1	2280.41		
719.3923	1	2600.75		

Formula Calculator Element Limits

Element	Min	Max
C	3	60
H	0	150
O	0	10

Formula Calculator Results

Formula	CalculatedMass	CalculatedMz	Mz	Diff. (mDa)	Diff. (ppm)	DBE
C41 H60 O5	632.4441	671.4072	671.4082	-1.00	-1.49	12.0000

--- End Of Report ---

Figure S2. HRESIMS spectrum of **1**.

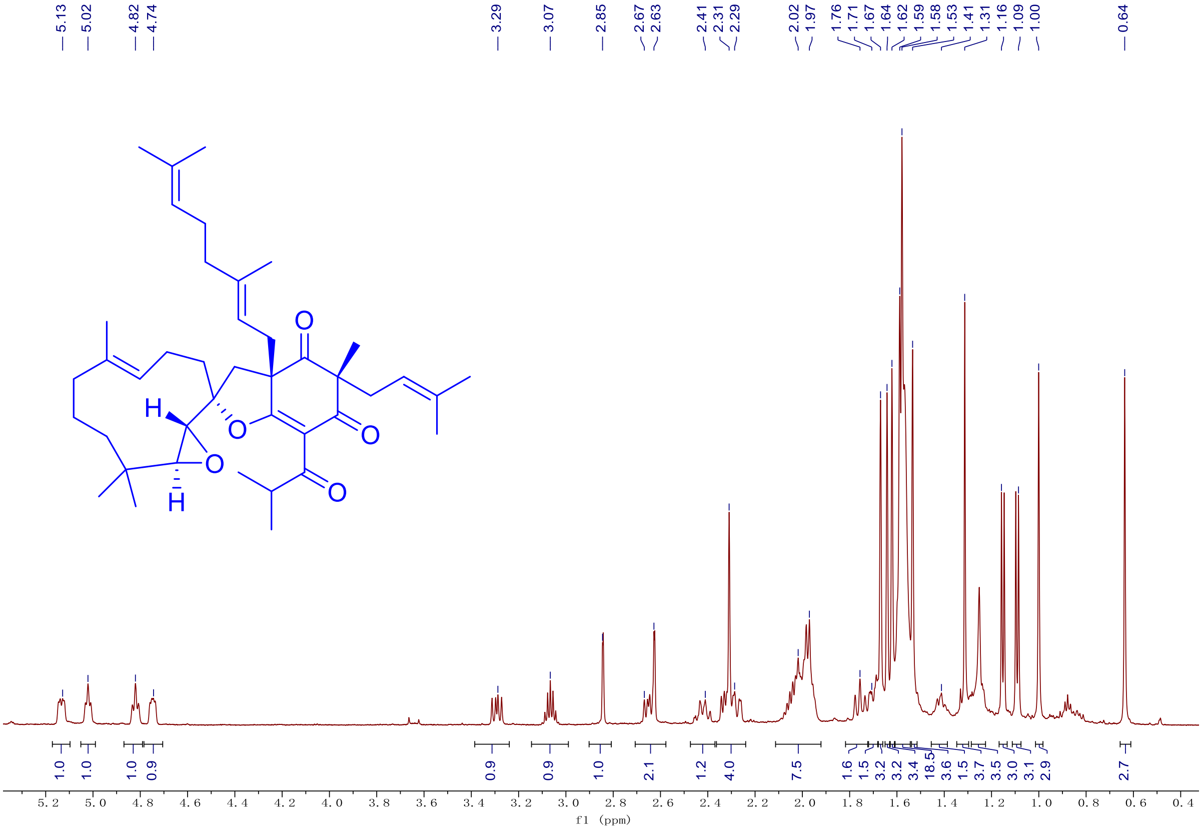

Figure S3. ^{1}H NMR spectrum of **1** (in $CDCl_3$, 600 MHz).

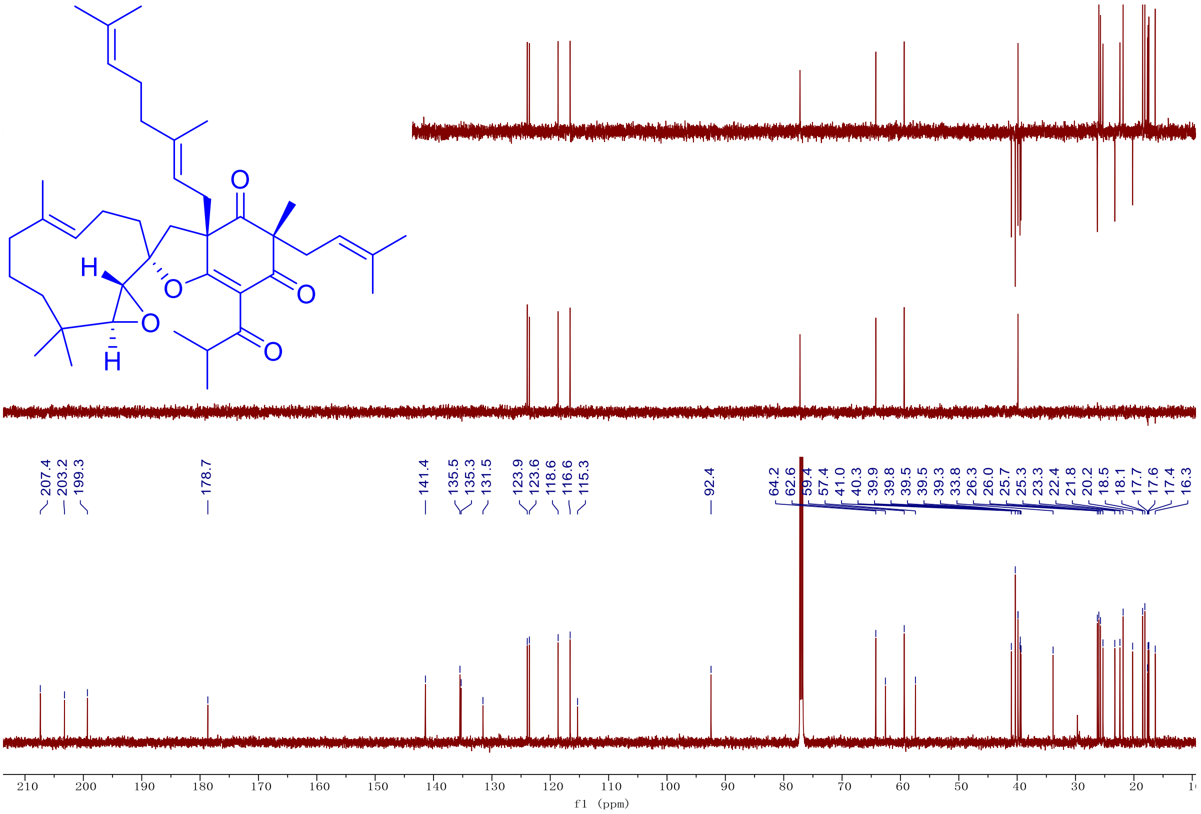

Figure S4. ^{13}C and DEPT NMR spectrum of **1** (in $CDCl_3$, 150 MHz).

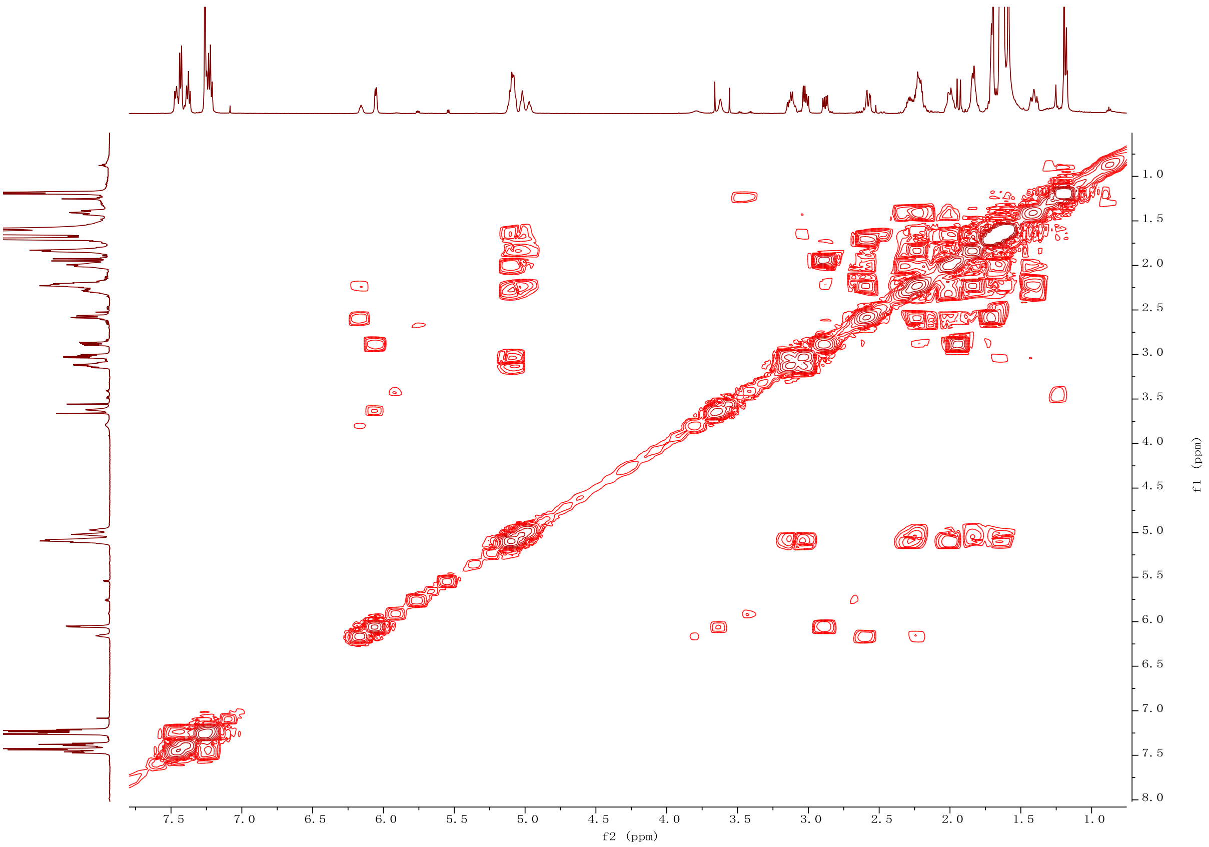

Figure S5. ^{1}H–^{1}H COSY spectrum of **1** (in $CDCl_3$, 600 MHz).

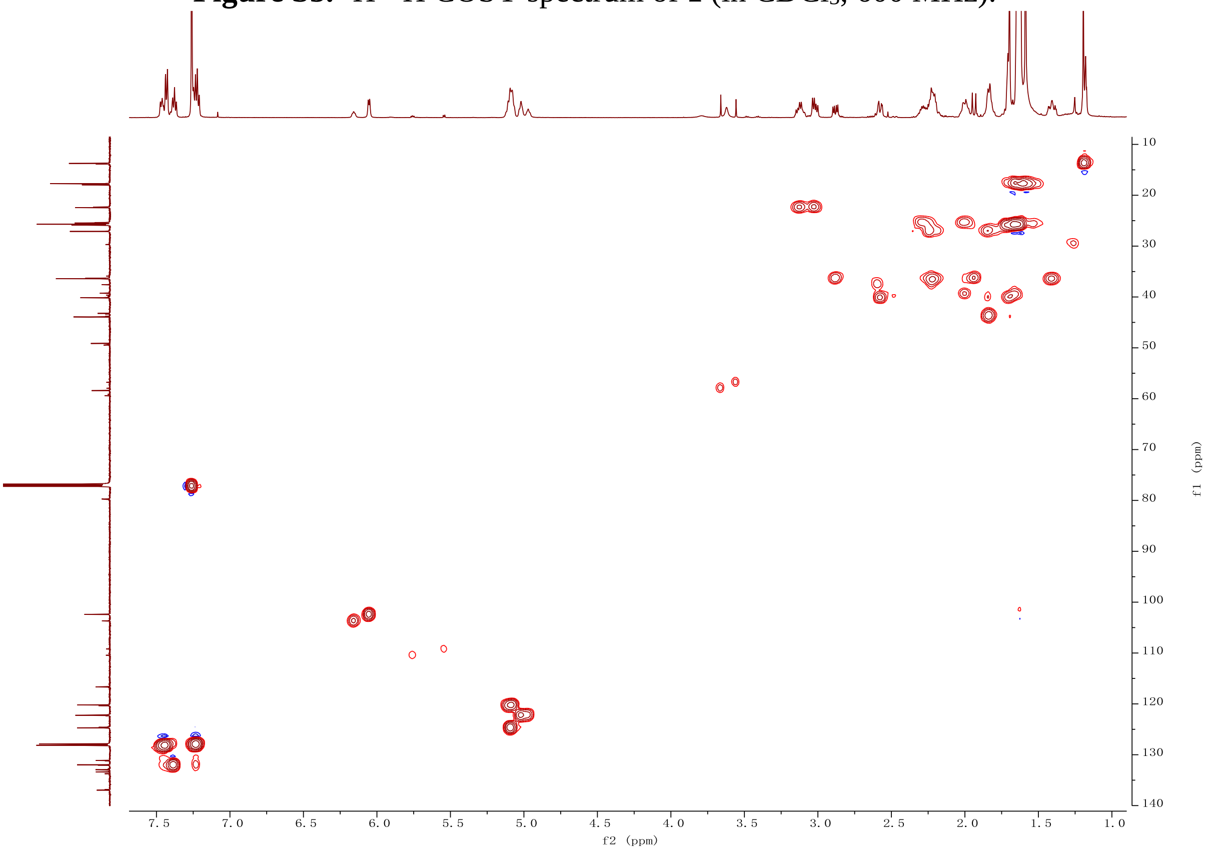

Figure S6. HSQC spectrum of **1** (in $CDCl_3$, 600 MHz).

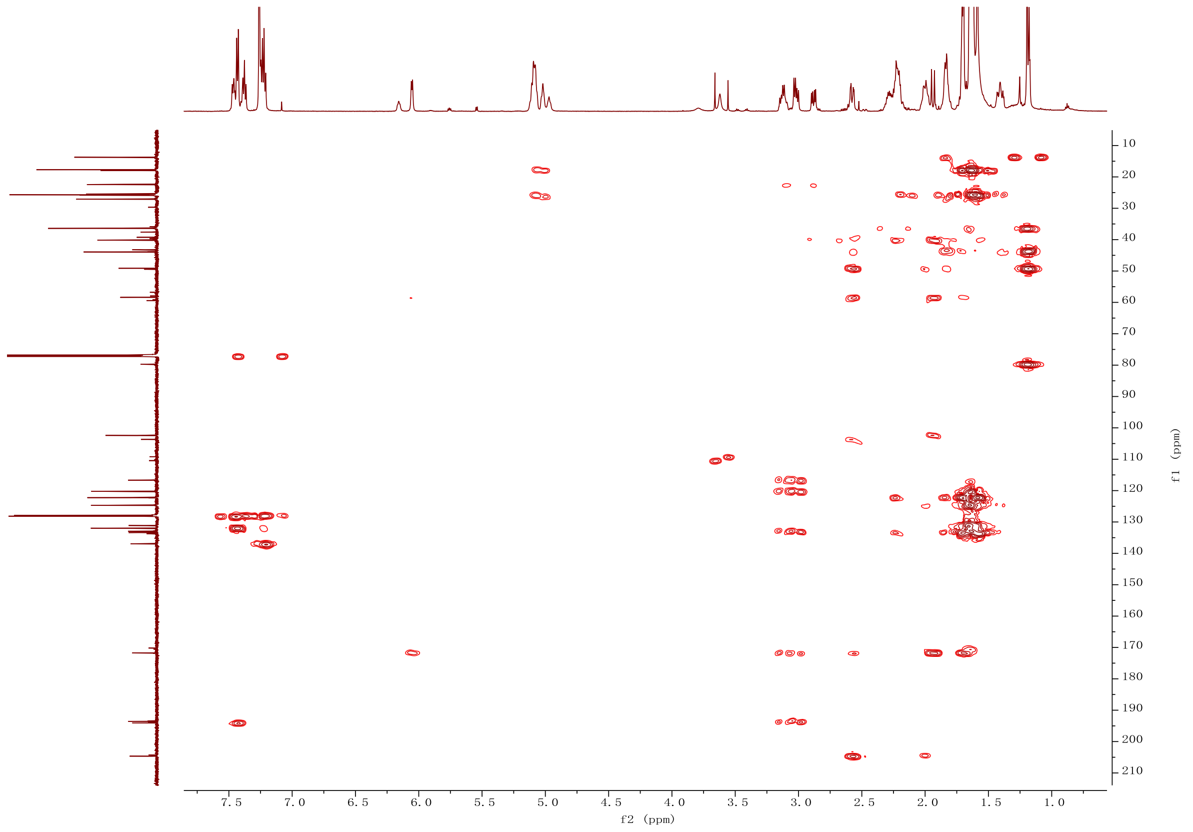

Figure S7. HMBC spectrum of compound **1** (in $CDCl_3$, 600 MHz).

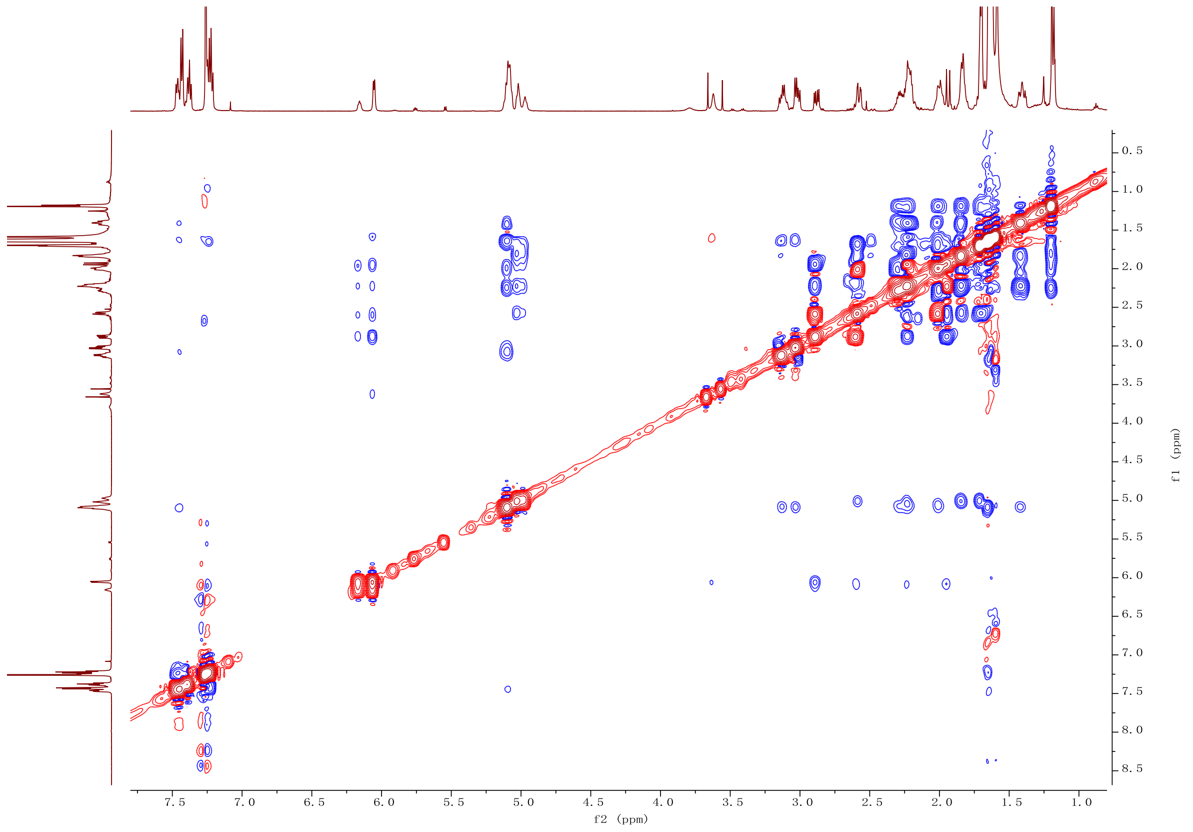

Figure S8. NOESY spectrum of **1** (in $CDCl_3$, 600 MHz).

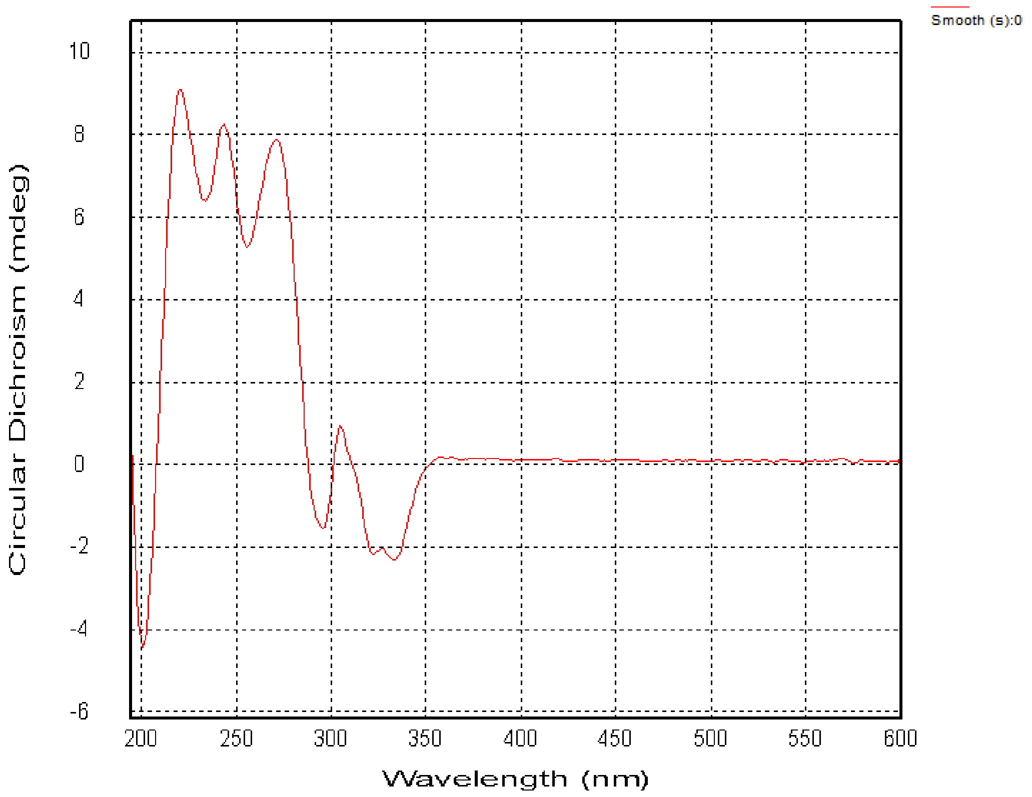

Figure S9. Experimental ECD of **2**.

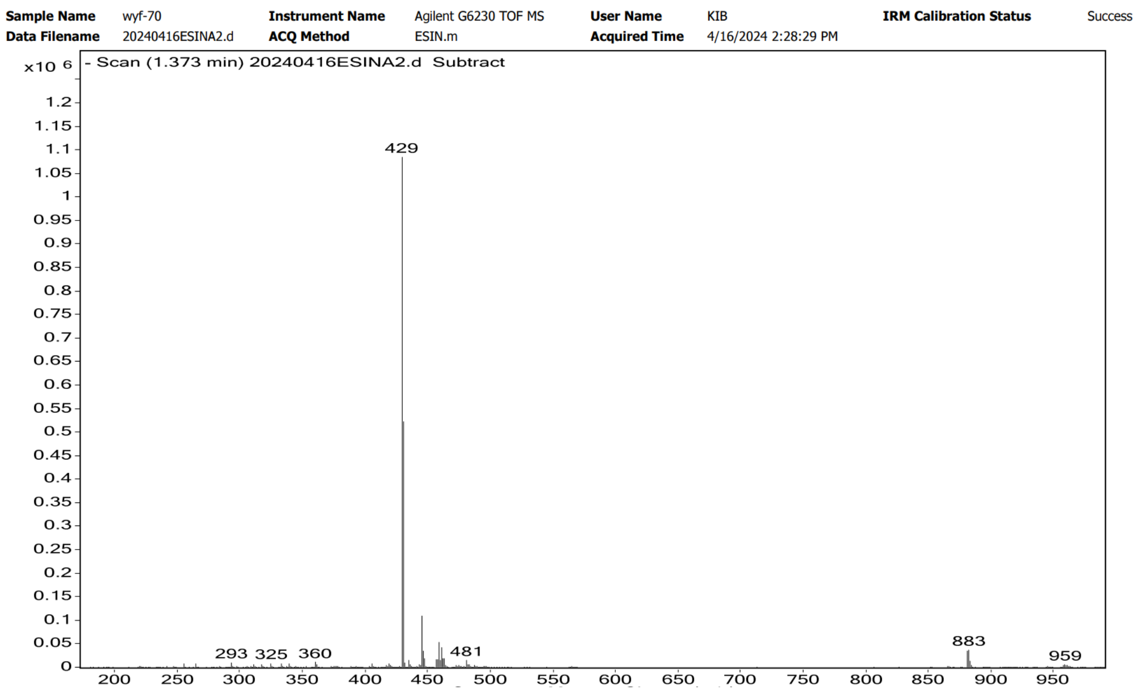

Figure S10. ESIMS spectrum of **2**.

Qualitative Analysis Report

**Data Filename**	20240416ESINA2.d	**Sample Name**	wyf-70
**Sample Type**	Sample	**Position**	
**Instrument Name**	Agilent G6230 TOF MS	**User Name**	KIB
**Acq Method**	ESIN.m	**Acquired Time**	4/16/2024 2:28:29 PM
**IRM Calibration Status**	Success	**DA Method**	ESI.m
**Comment**			

**Sample Group**		**Info.**
**Acquisition SW Version**	6200 series TOF/6500 series Q-TOF B.05.01 (B5125.2)	

User Spectra

Fragmentor Voltage	Collision Energy	Ionization Mode
200	0	ESI

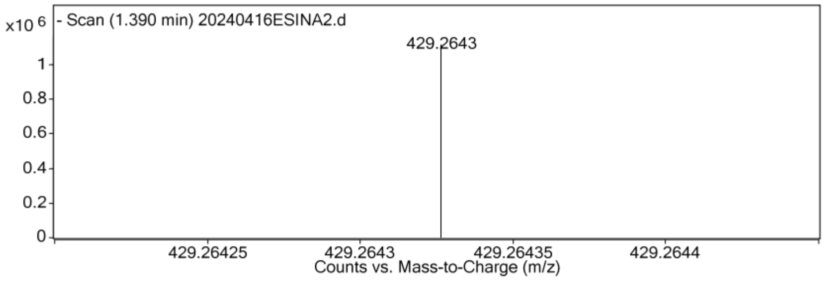

Peak List

m/z	z	Abund	Formula	Ion
429.2643	1	1111459.25	C26 H37 O5	M-
430.2695	1	533873.31	C26 H37 O5	M-
1033.9881	1	374013.41		
1311.7755		114112.13		
1312.7809	1	215744.22		
1313.7839	1	188658.34		
1327.7549		325197.94		
1328.7597	1	522958		
1329.764	1	385134.56		
1330.7669	1	175542.97		

Formula Calculator Element Limits

Element	Min	Max
C	0	200
H	0	400
O	0	10

Formula Calculator Results

Formula	CalculatedMass	Mz	Diff.(mDa)	Diff. (ppm)	DBE
C26 H37 O5	429.2641	429.2643	-0.2	0.5	8.5

--- End Of Report ---

Figure S11. HRESIMS spectrum of **2**.

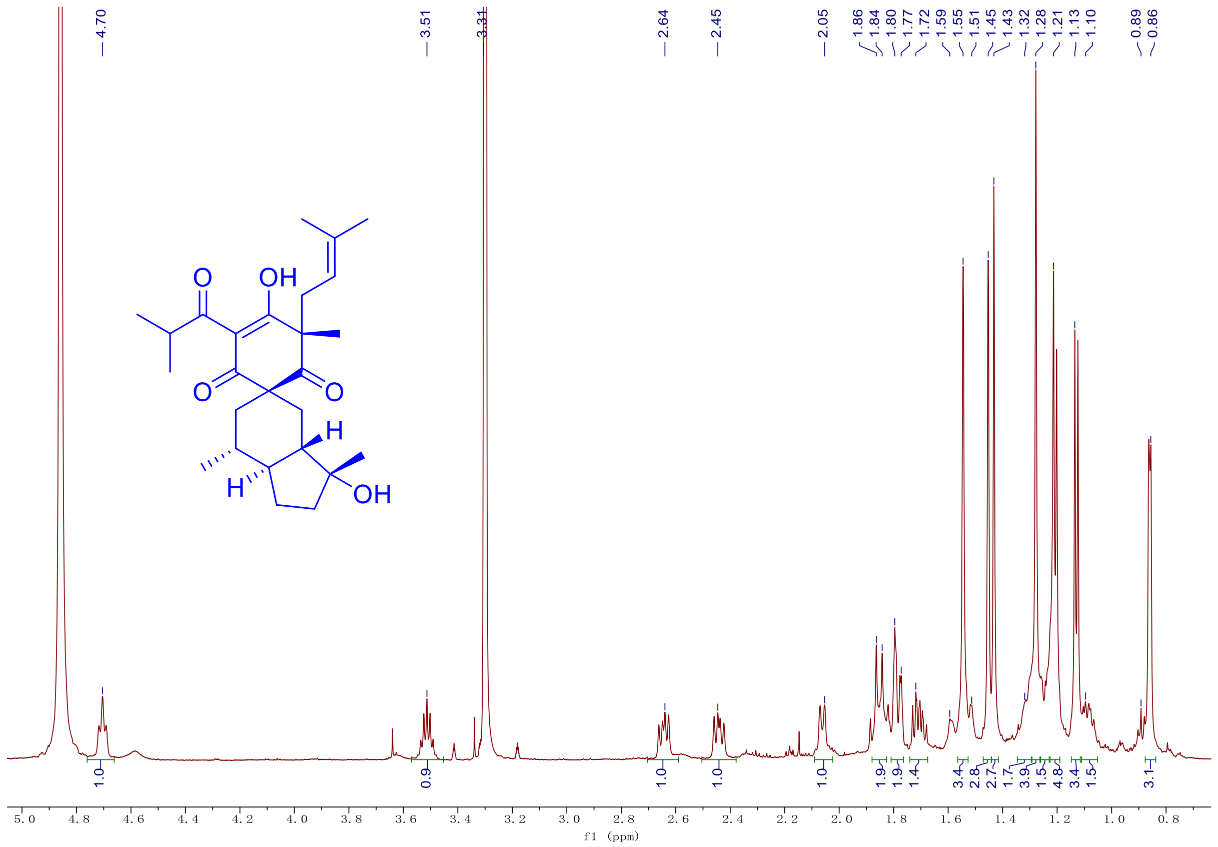

Figure S12. ^{1}H NMR spectrum of **2** (in CD_3OD, 600 MHz).

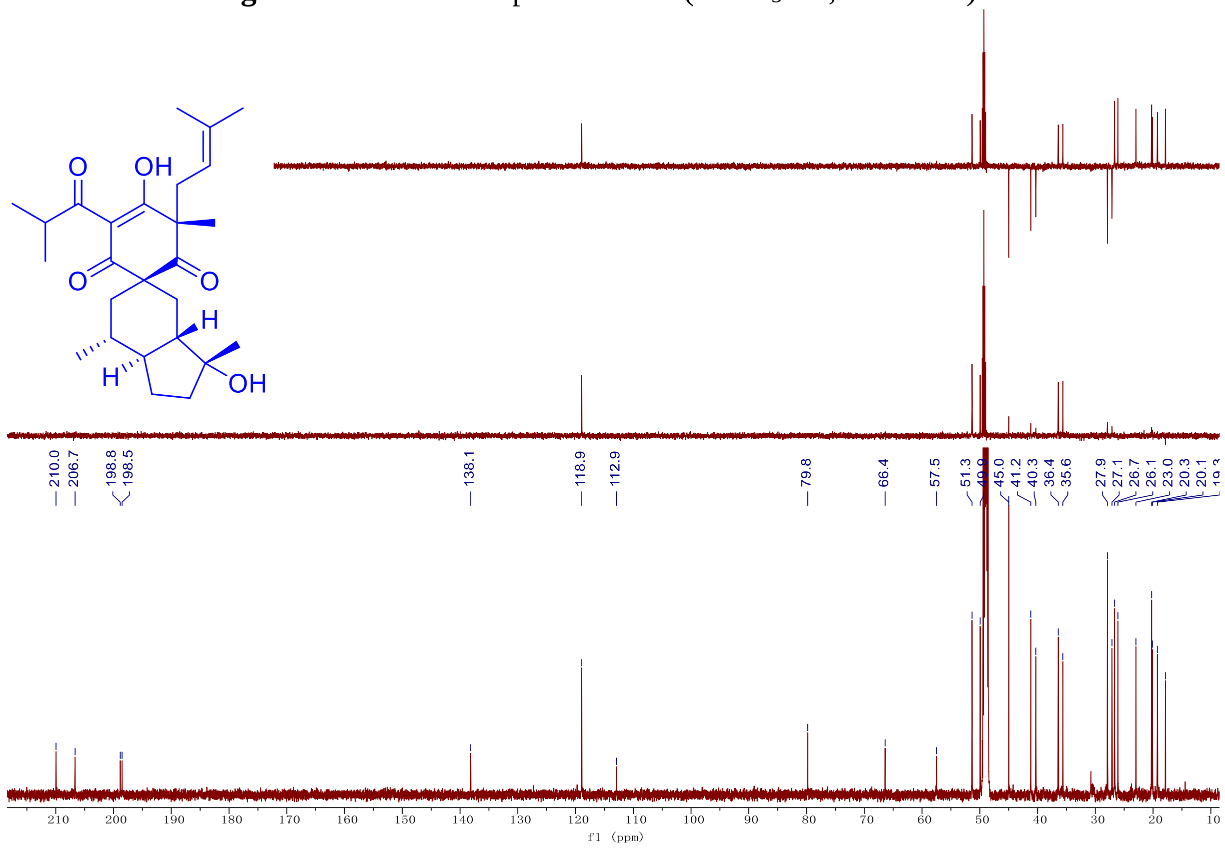

Figure S13. ^{13}C and DEPT NMR spectrum of **2** (in CD_3OD, 150 MHz).

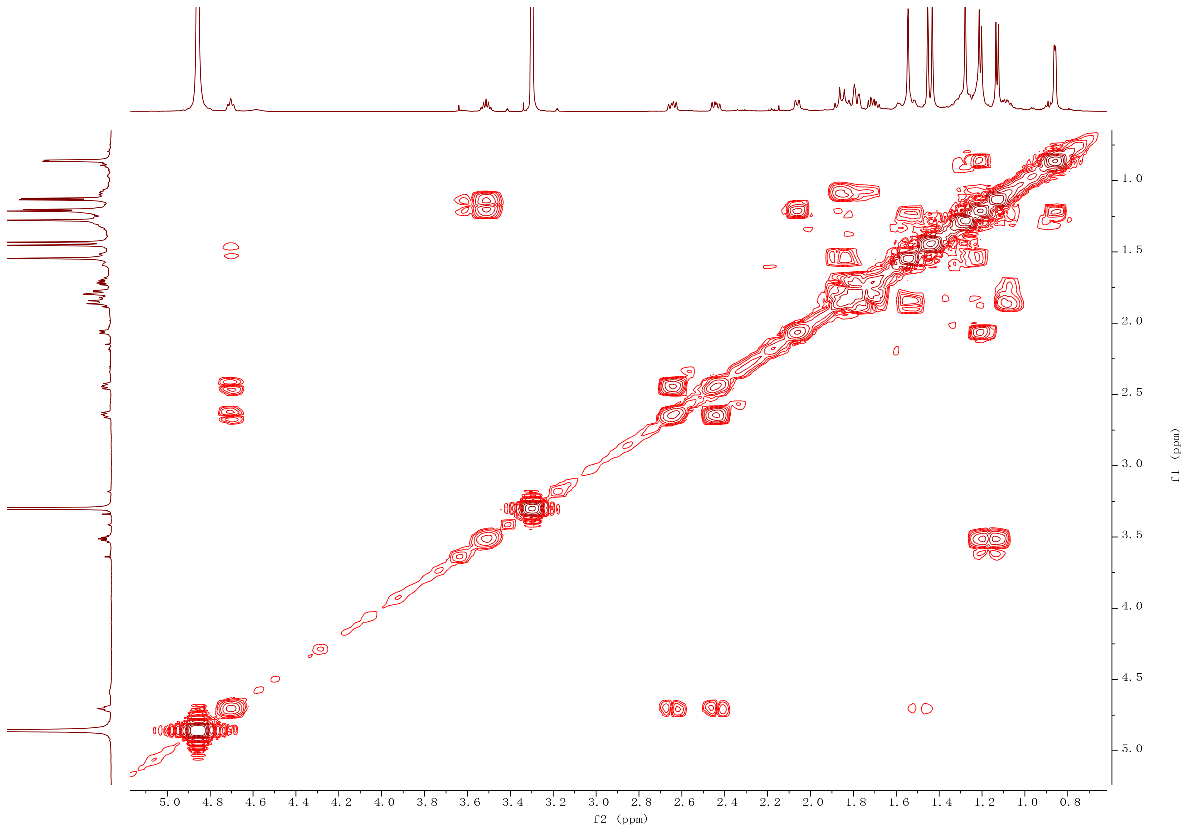

Figure S14. ¹H–¹H COSY spectrum of **2** (in CD_3OD, 600 MHz).

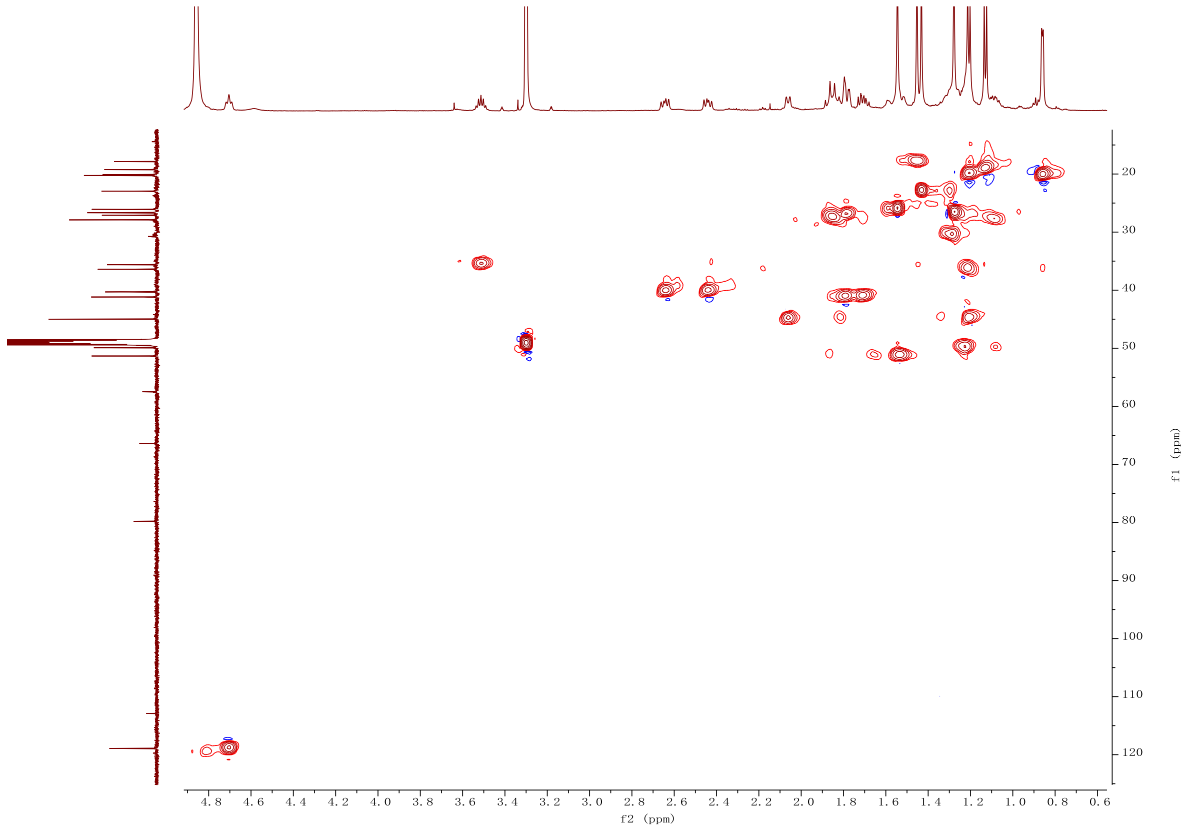

Figure S15. HSQC spectrum of **2** (in CD_3OD, 600 MHz).

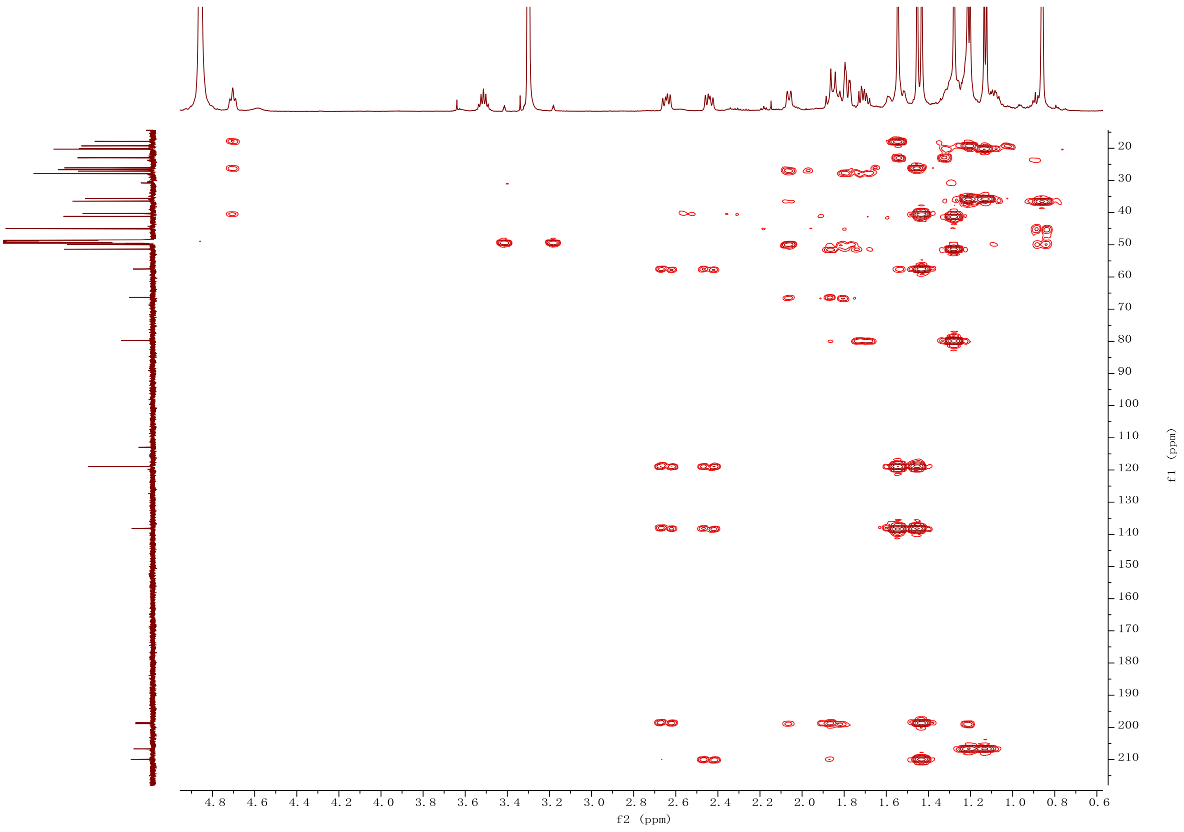

Figure S16. HMBC spectrum of **2** (in CD_3OD, 600 MHz).

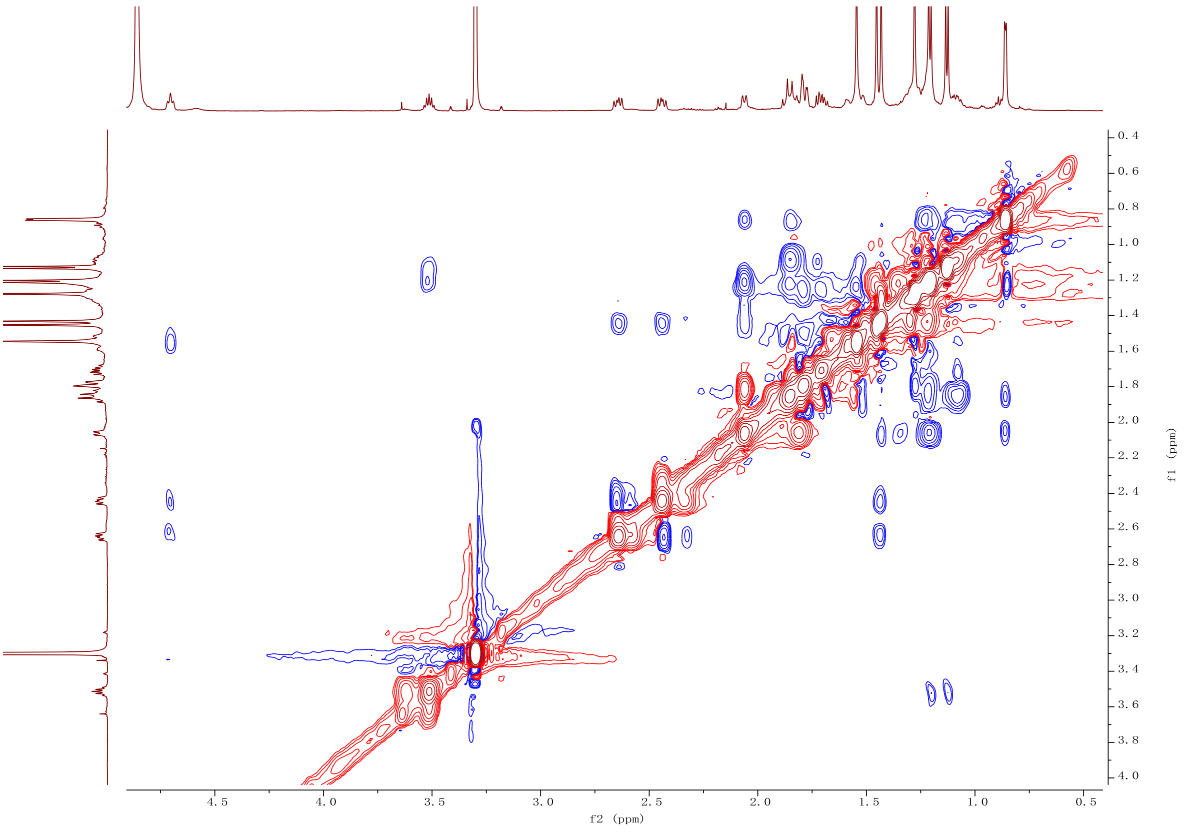

Figure S19. NOESY spectrum of **2** (in CD_3OD, 600 MHz).

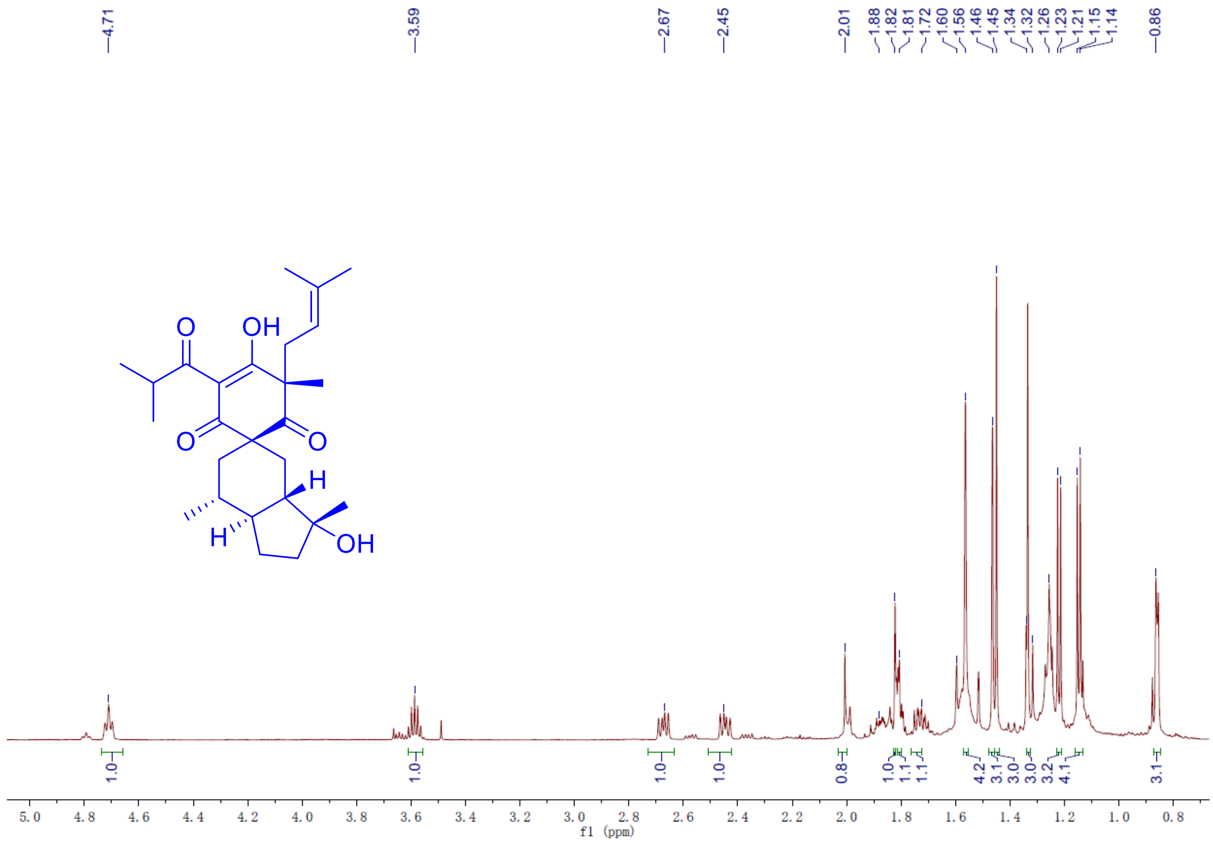

Figure S20. ^{1}H NMR spectrum of **2** (in $CDCl_3$, 600 MHz).

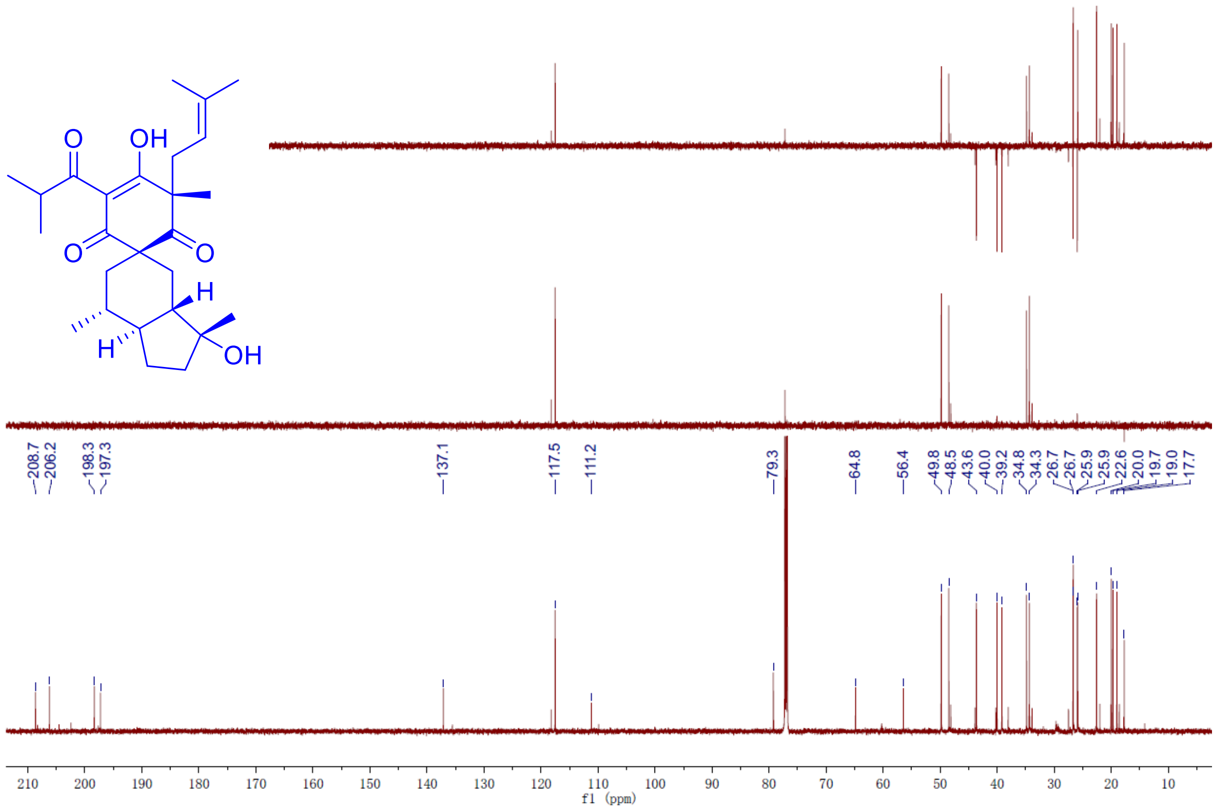

Figure S21. ^{13}C and DEPT NMR spectrum of **2** (in $CDCl_3$, 150 MHz).

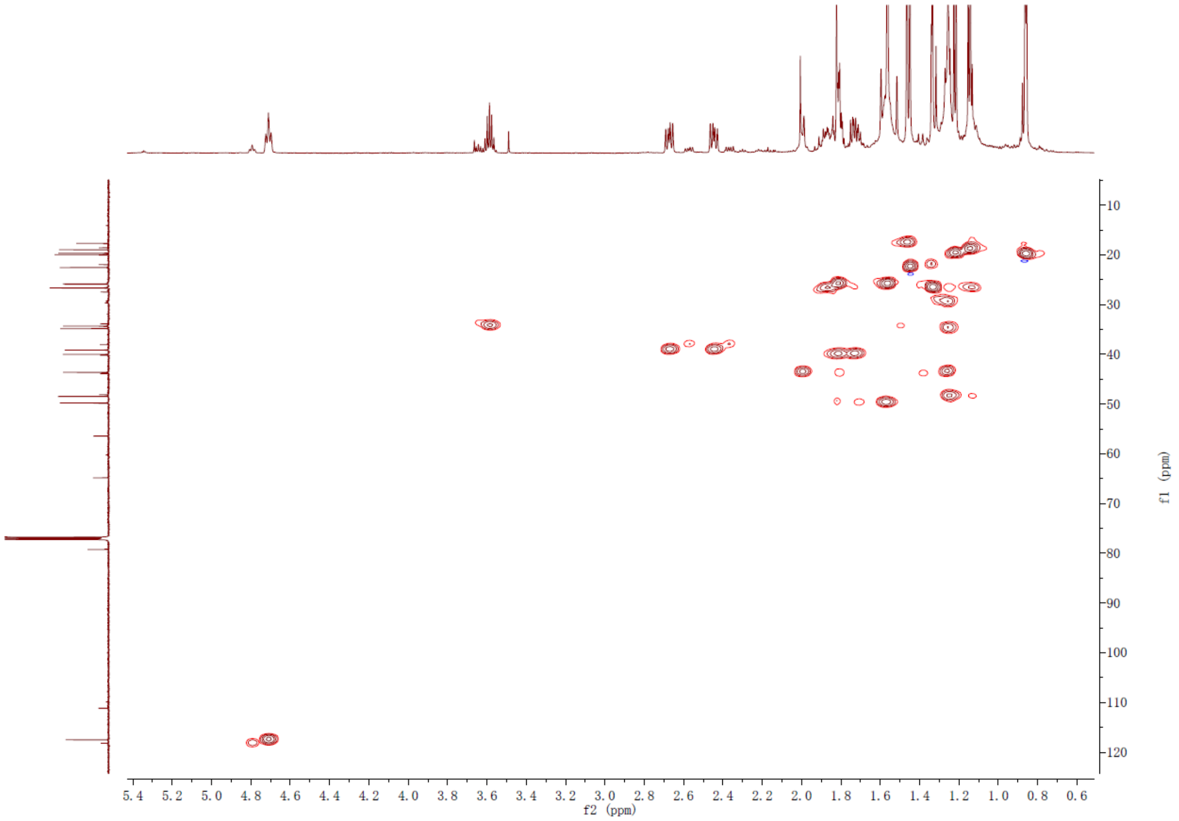

Figure S22. HSQC spectrum of **2** (in $CDCl_3$, 600 MHz).

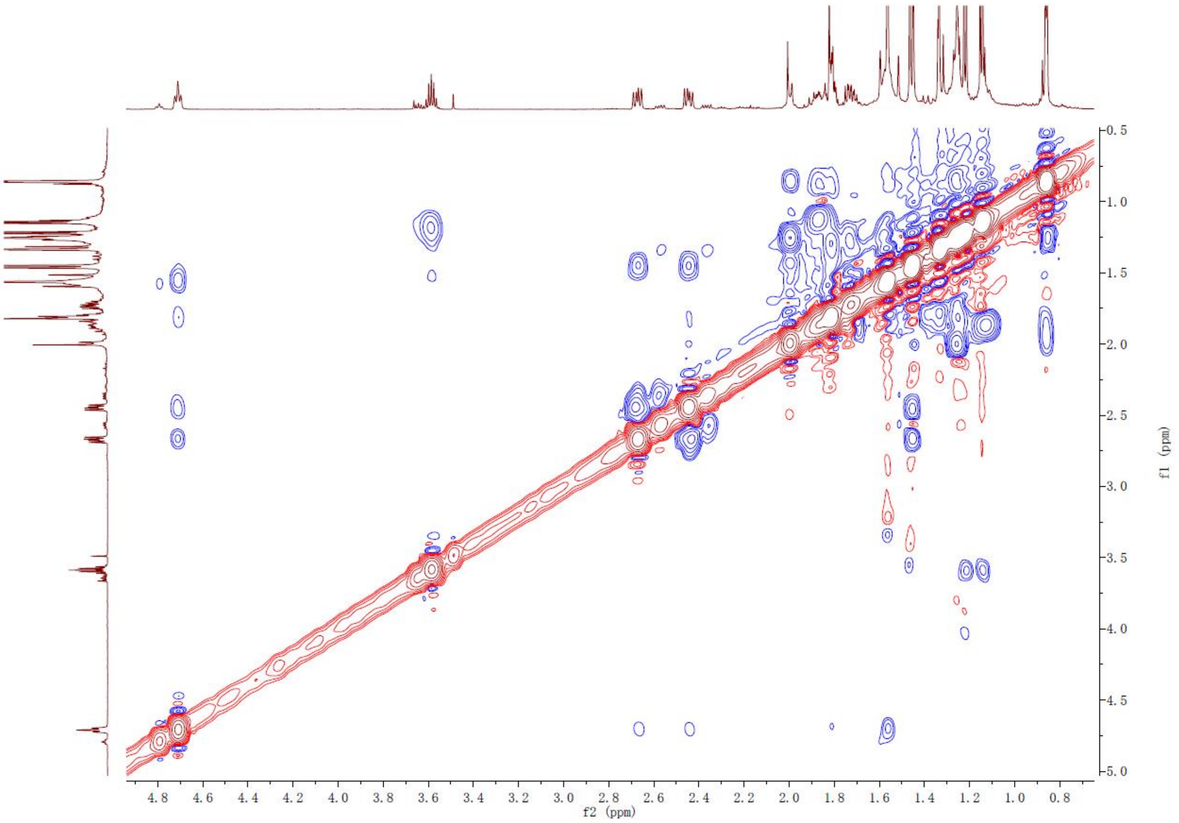

Figure S23. NOESY spectrum of **2** (in $CDCl_3$, 600 MHz).

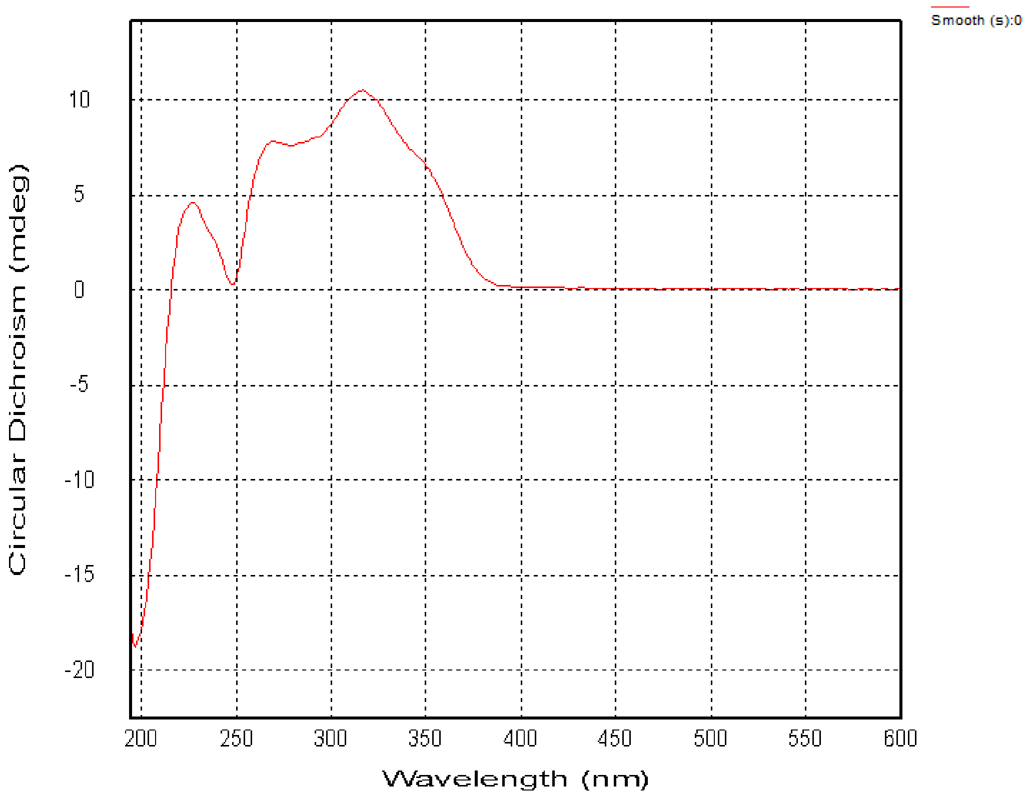

Figure S24. Experimental ECD of **3**.

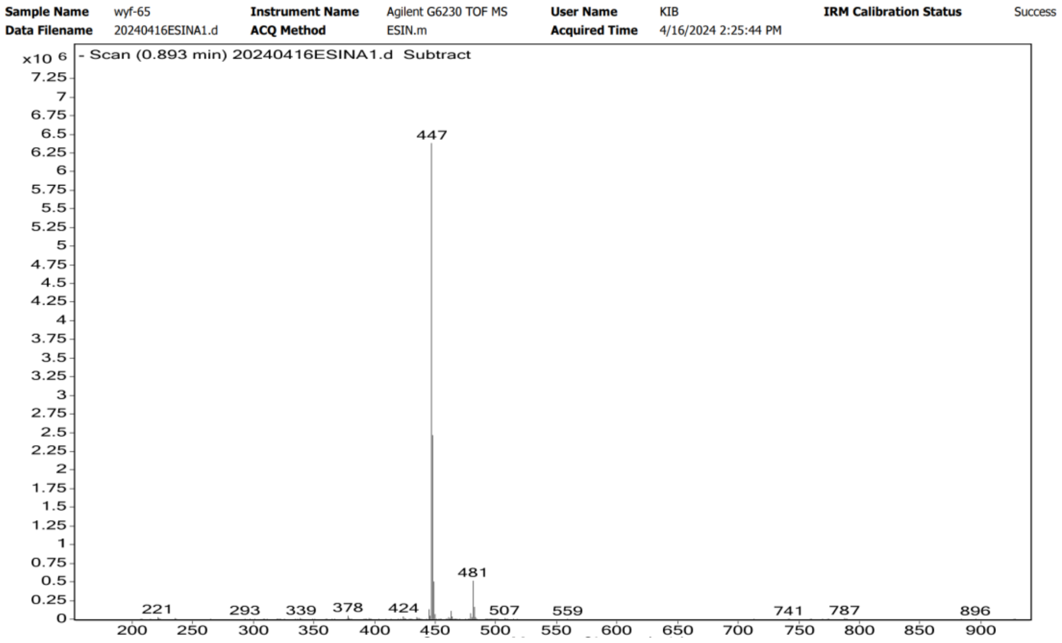

Figure S25. ESIMS spectrum of **3**.

Qualitative Analysis Report

**Data Filename**	20240416ESINA1.d	**Sample Name**	wyf-65
**Sample Type**	Sample	**Position**	
**Instrument Name**	Agilent G6230 TOF MS	**User Name**	KIB
**Acq Method**	ESIN.m	**Acquired Time**	4/16/2024 2:25:44 PM
**IRM Calibration Status**	Success	**DA Method**	ESI.m
**Comment**			

**Sample Group**		**Info.**
**Acquisition SW Version**	6200 series TOF/6500 series Q-TOF B.05.01 (B5125.2)	

User Spectra

Fragmentor Voltage	Collision Energy	Ionization Mode
200	0	ESI

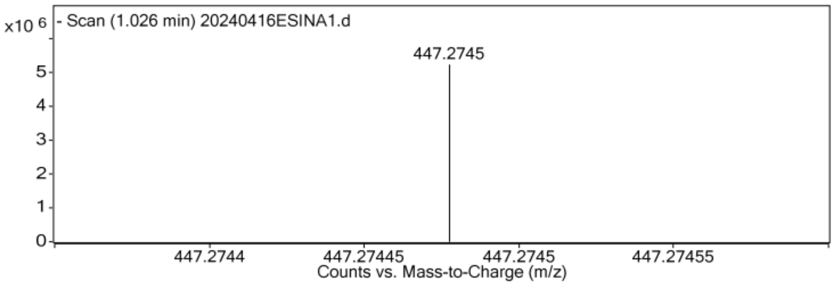

Peak List

m/z	z	Abund	Formula	Ion
445.2585	1	103201.79		
447.2745	1	5218060.5	C26 H39 O6	M-
447.4648	1	288698.59		
448.2785	1	2013364.5	C26 H39 O6	M-
448.4704	1	115402.41		
449.2825	1	399319.59	C26 H39 O6	M-
463.2687	1	84434.41		
481.2801	1	414341.56		
482.2836	1	126001.45		
1033.9881	1	299942.91		

Formula Calculator Element Limits

Element	Min	Max
C	0	200
H	0	400
O	0	10

Formula Calculator Results

Formula	CalculatedMass	Mz	Diff.(mDa)	Diff. (ppm)	DBE
C26 H39 O6	447.2747	447.2745	0.2	0.4	7.5

--- End Of Report ---

Figure S26. HRESIMS spectrum of **3**.

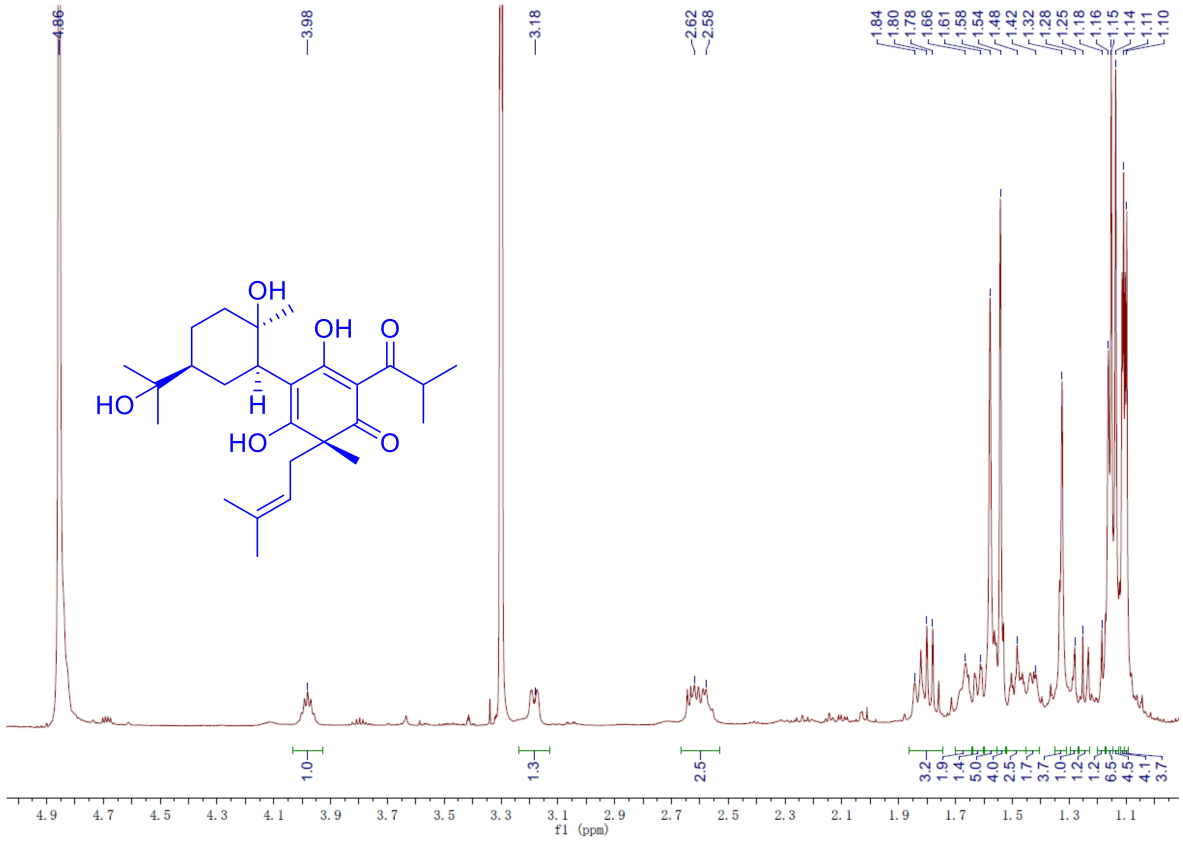

Figure S27. ^{1}H NMR spectrum of **3** (in CD_3OD, 600 MHz).

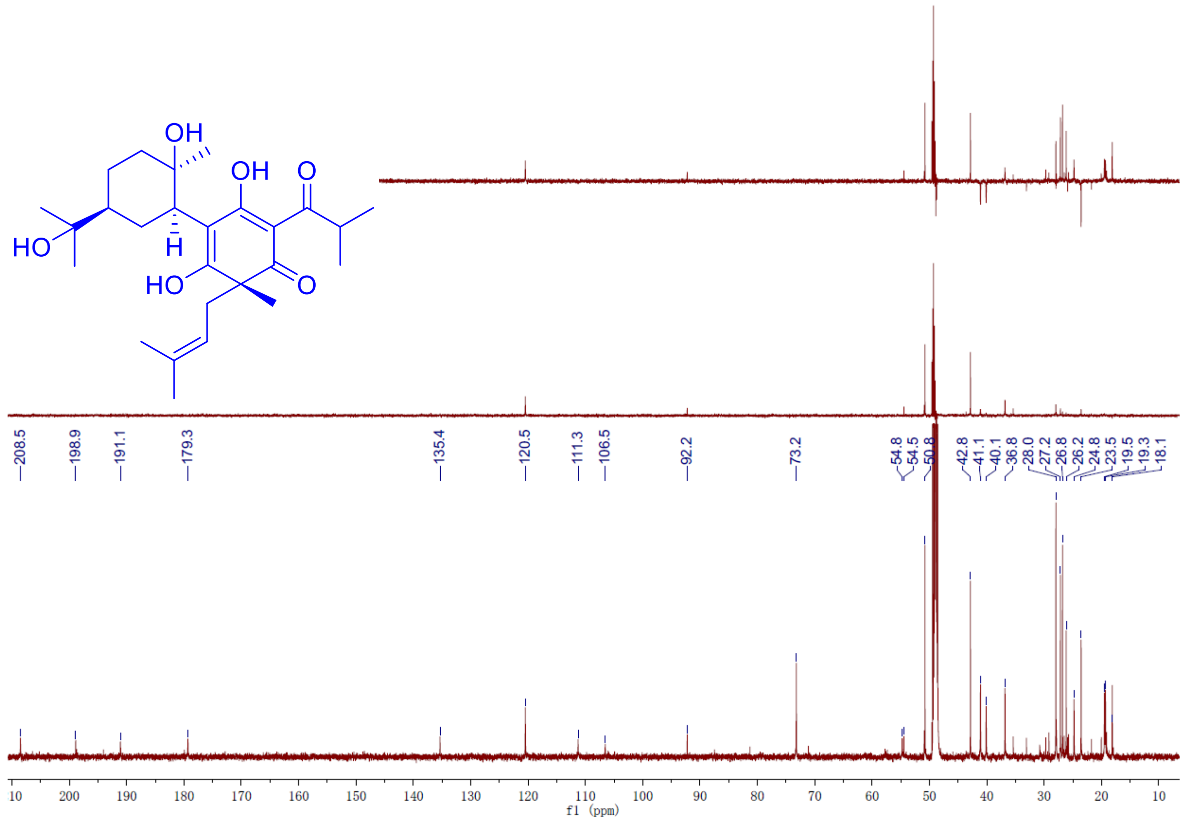

Figure S28. ^{13}C and DEPT NMR spectrum of **3** (in CD_3OD, 150 MHz).

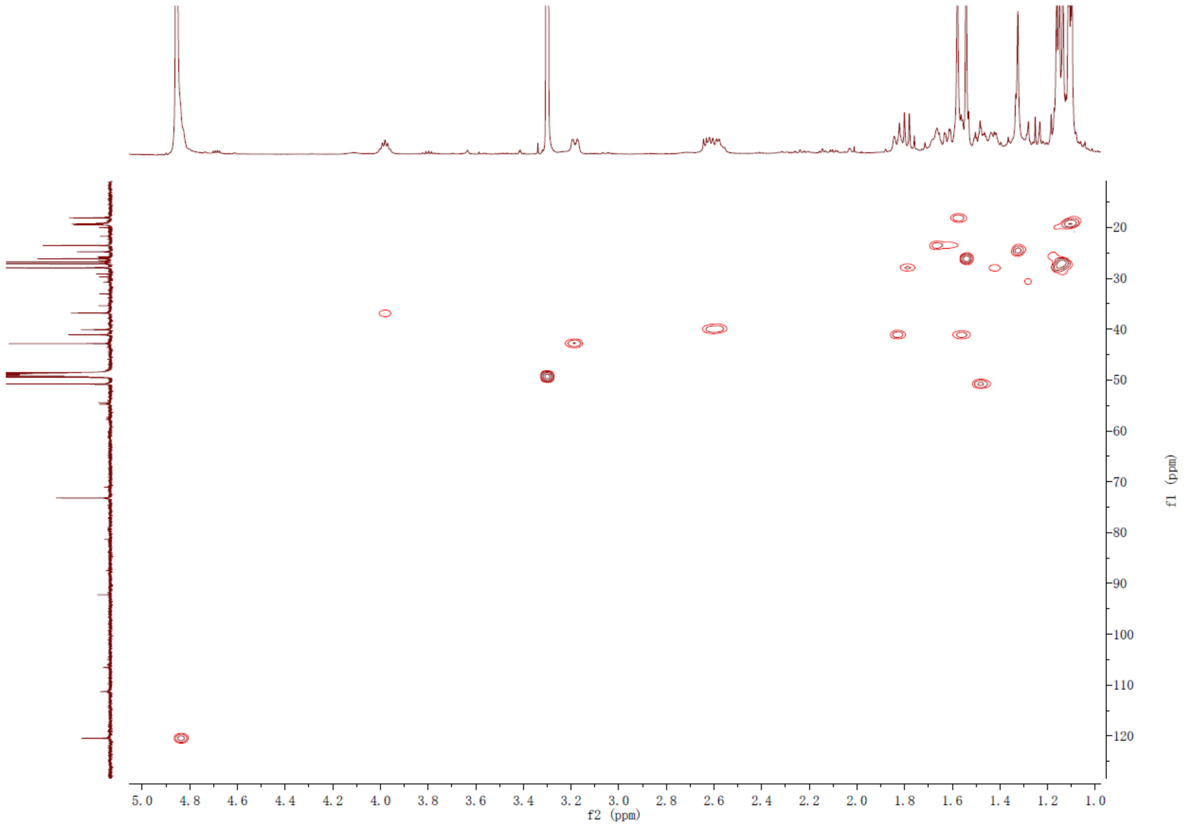

Figure S29. The HSQC spectrum of **3** (in CD3OD, 600 MHz).

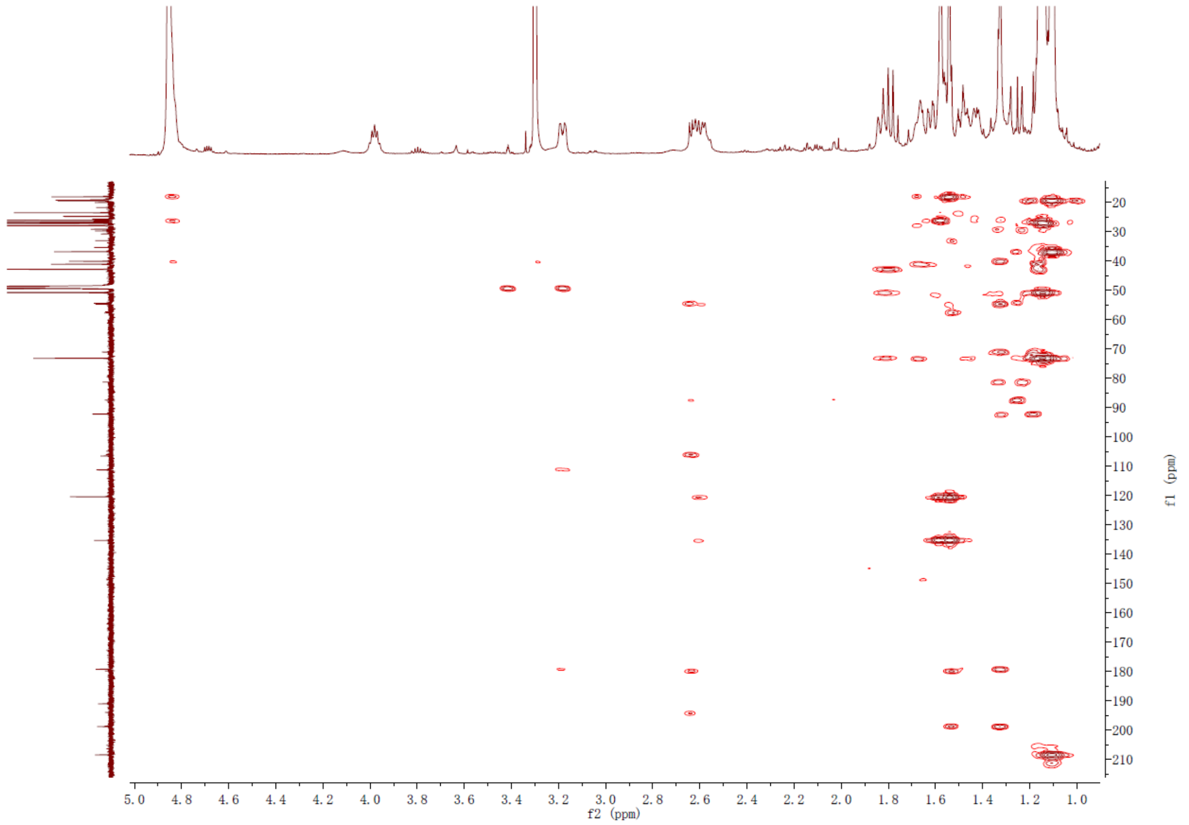

Figure S30. HMBC spectrum of **3** (in CD3OD, 600 MHz).

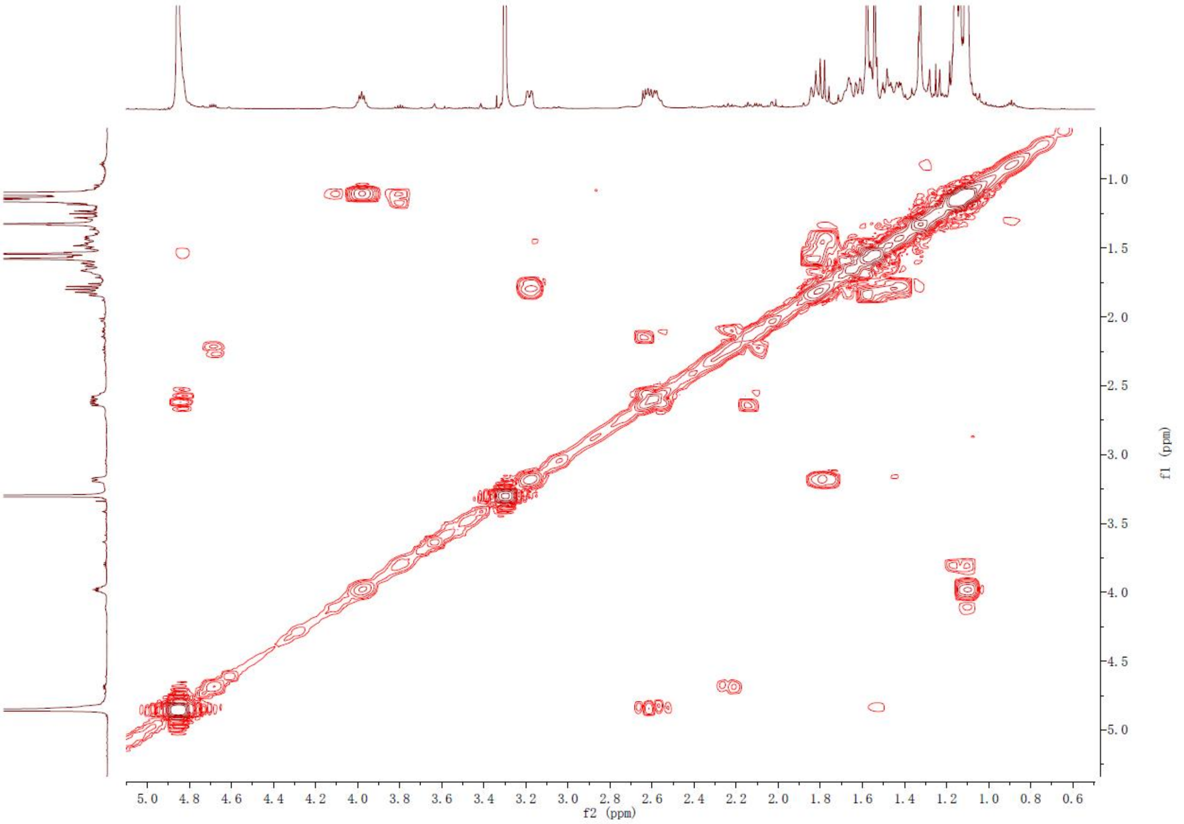

Figure S31. ^{1}H–^{1}H COSY spectrum of **3** (in CD_3OD, 600 MHz).

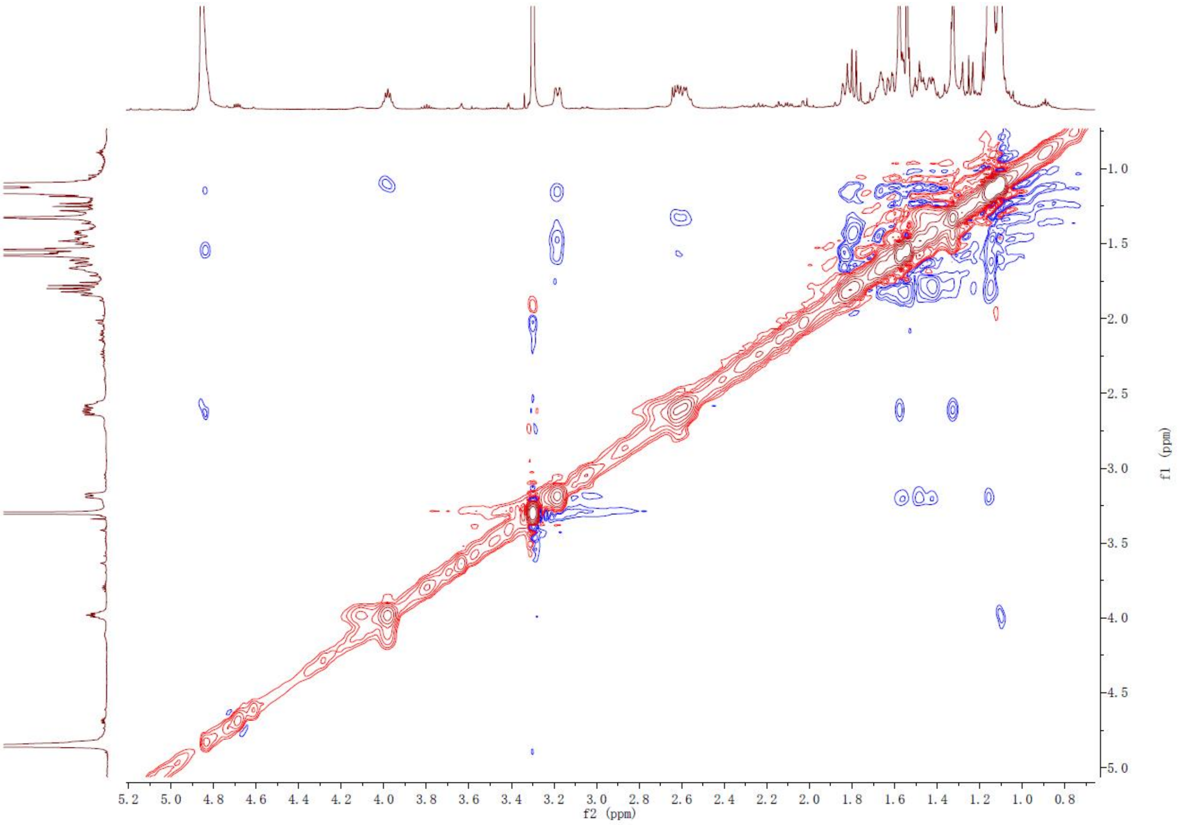

Figure S32. NOESY spectrum of **3** (in CD_3OD, 600 MHz).

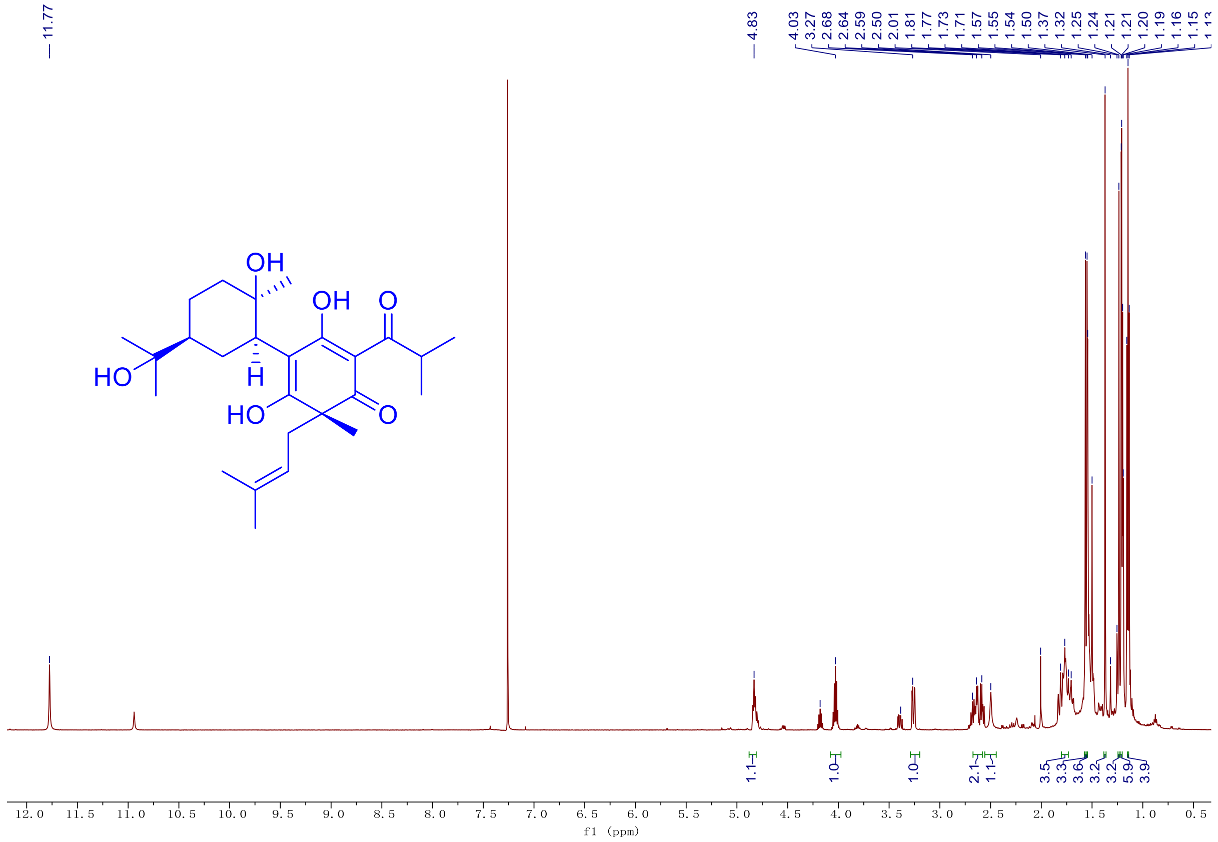

Figure S33. ^{1}H NMR spectrum of **3** (in $CDCl_3$, 600 MHz).

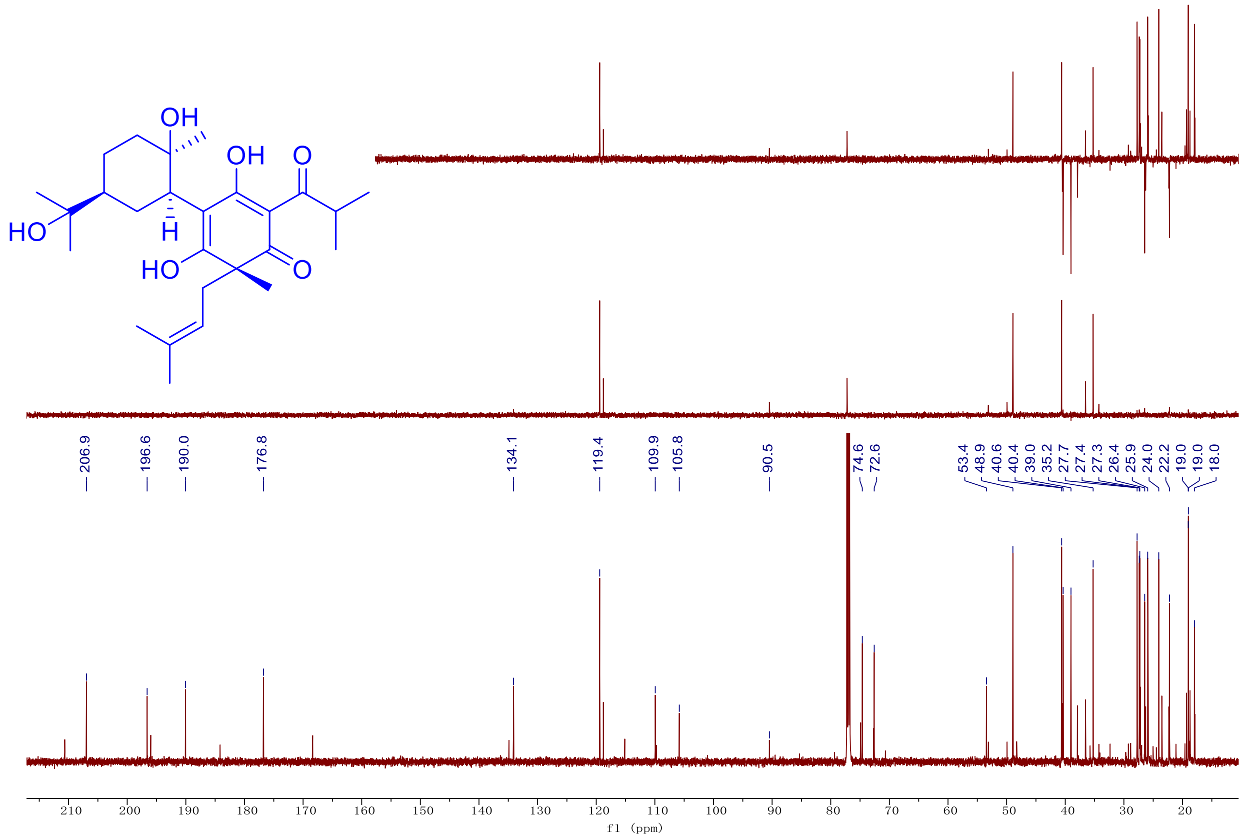

Figure S34. ^{13}C NMR spectrum of **3** (in $CDCl_3$, 150 MHz).

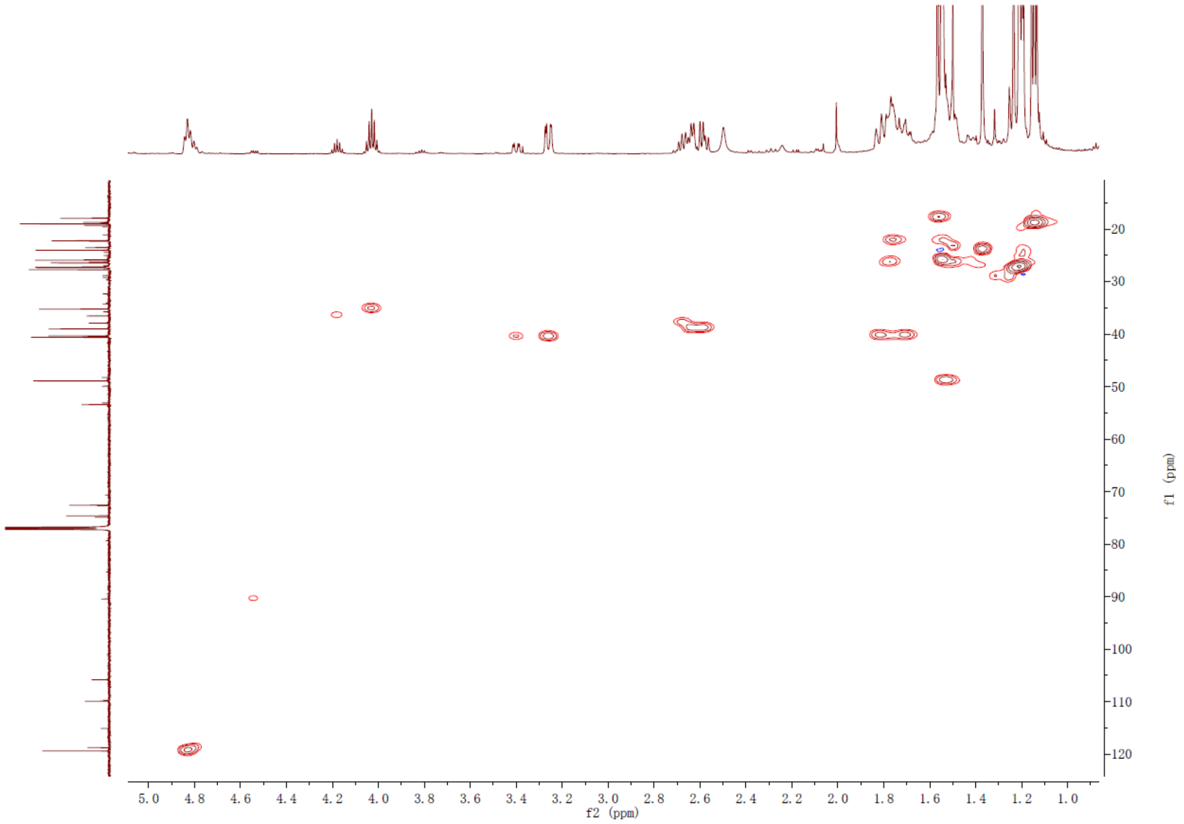

Figure S35. HSQC spectrum of **3** (in $CDCl_3$, 600 MHz).

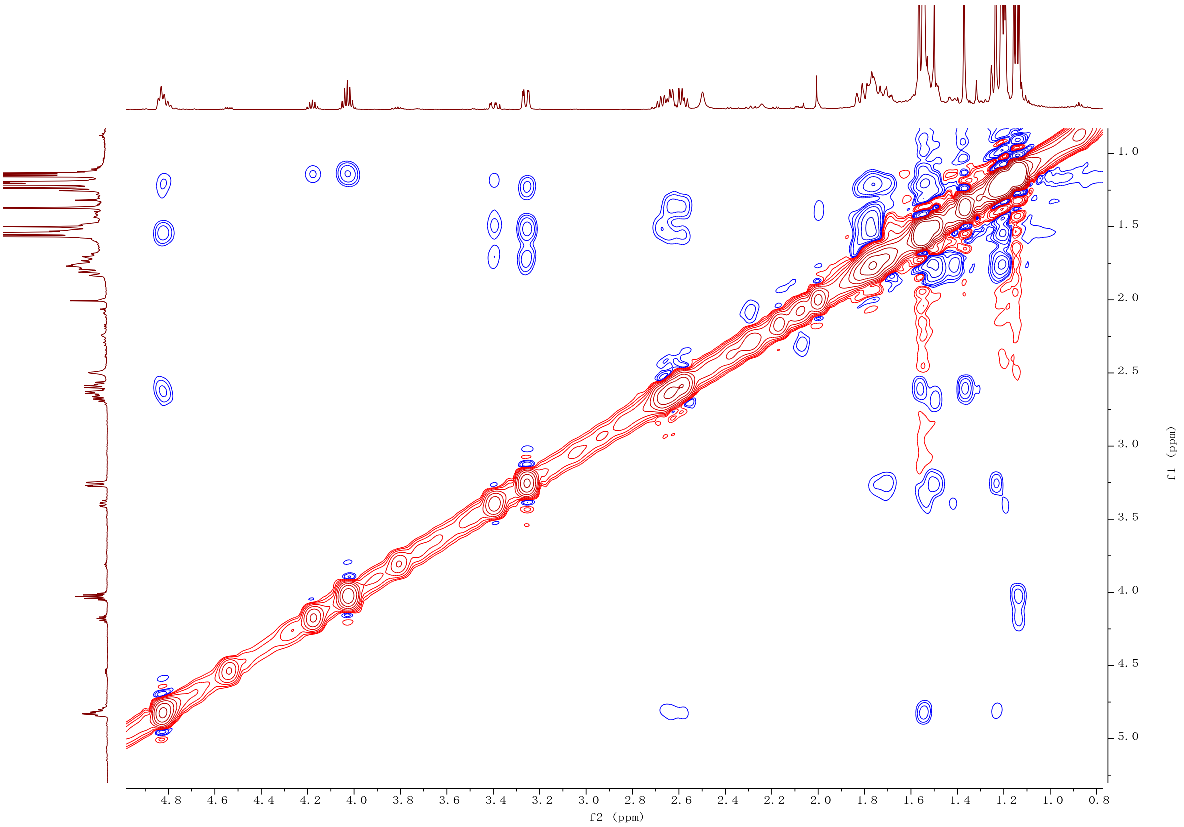

Figure S36. NOESY spectrum of **3** (in $CDCl_3$, 600 MHz).

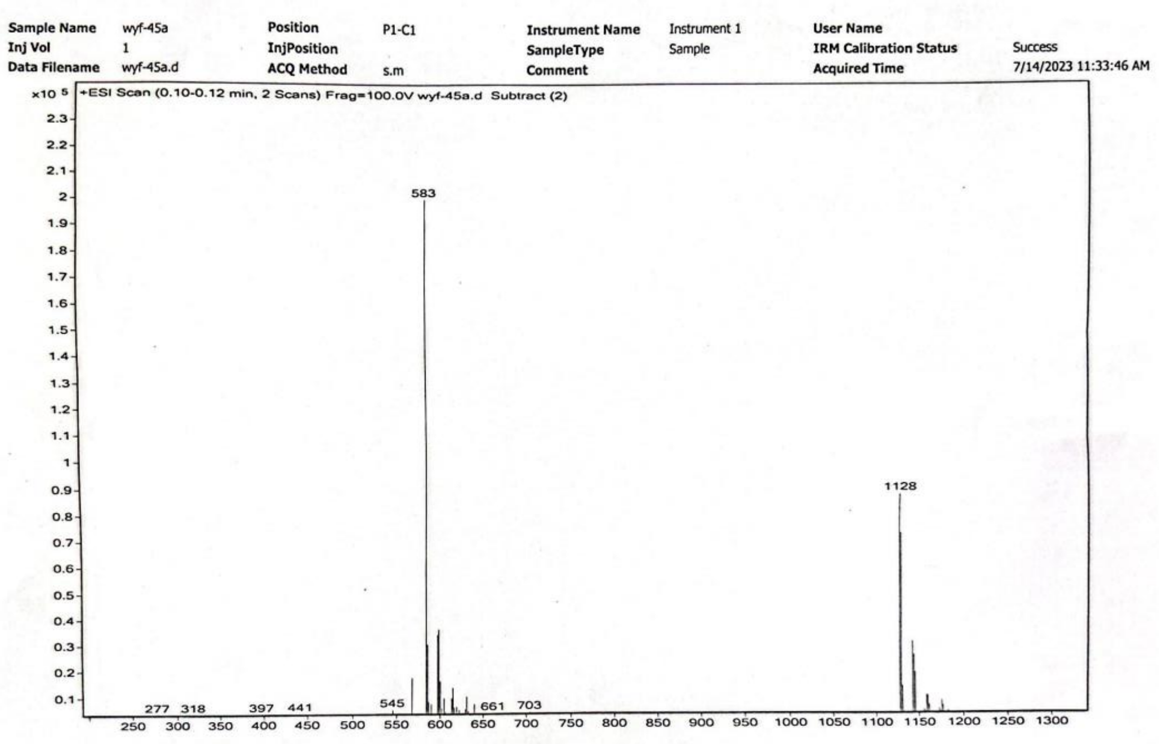

Figure S37. ESIMS spectrum of **4**.

Qualitative Analysis Report

**Data Filename**	wyf-45a.d	**Sample Name**	wyf-45a
**Sample Type**	Sample	**Position**	P1-C1
**Instrument Name**	Instrument 1	**User Name**	
**Acq Method**	s.m	**Acquired Time**	7/14/2023 11:33:46 AM
**IRM Calibration Status**	Success	**DA Method**	PCDL.m
**Comment**			

Sample Group **Info.**

Acquisition SW Version 6200 series TOF/6500 series Q-TOF B.05.01 (B5125.2)

User Spectra

Fragmentor Voltage	Collision Energy	Ionization Mode
100	0	ESI

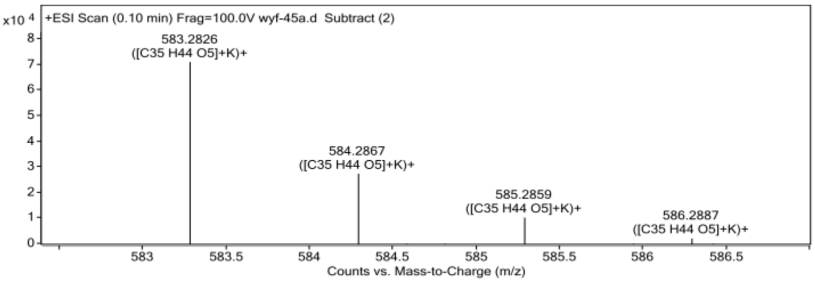

Peak List

m/z	z	Abund	Formula	Ion
583.2826	1	71120.25	C35 H44 O5	(M+K)+
584.2867	1	27505.93	C35 H44 O5	(M+K)+
585.2859	1	10456.64	C35 H44 O5	(M+K)+
597.2965	1	14562.8		
599.2808	1	14776.93		
1127.602	1	27157.26		
1128.6053	1	21075.87		
1129.6016	1	10148.93		
1141.6182	1	9515.27		
1142.6174	1	8802.46		

Formula Calculator Element Limits

Element	Min	Max
C	3	60
H	0	150
O	0	10

Formula Calculator Results

Formula	CalculatedMass	CalculatedMz	Mz	Diff. (mDa)	Diff. (ppm)	DBE
C35 H44 O5	544.3189	583.2820	583.2826	-0.60	-1.03	14.0000

--- End Of Report ---

Figure S38. HRESIMS spectrum of **4**.

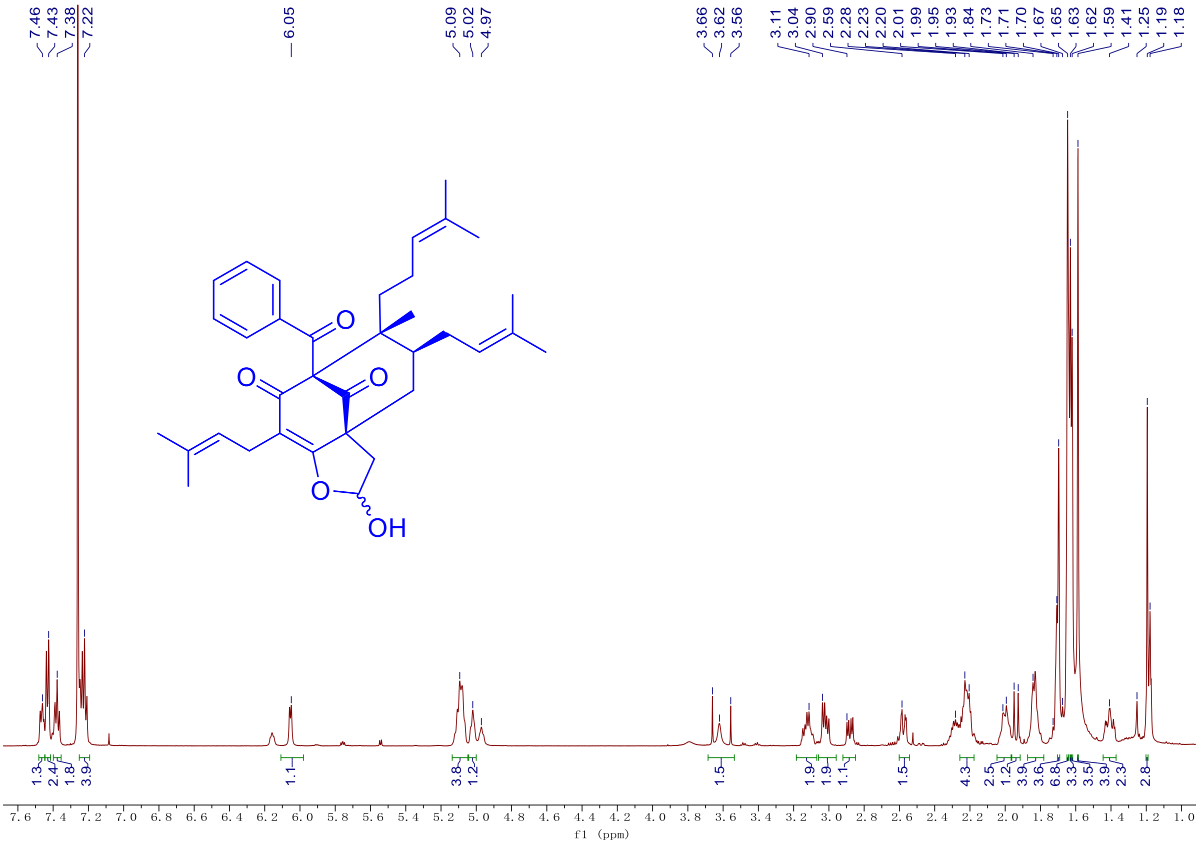

Figure S39. ^{1}H NMR spectrum of **4** (in $CDCl_3$, 600 MHz).

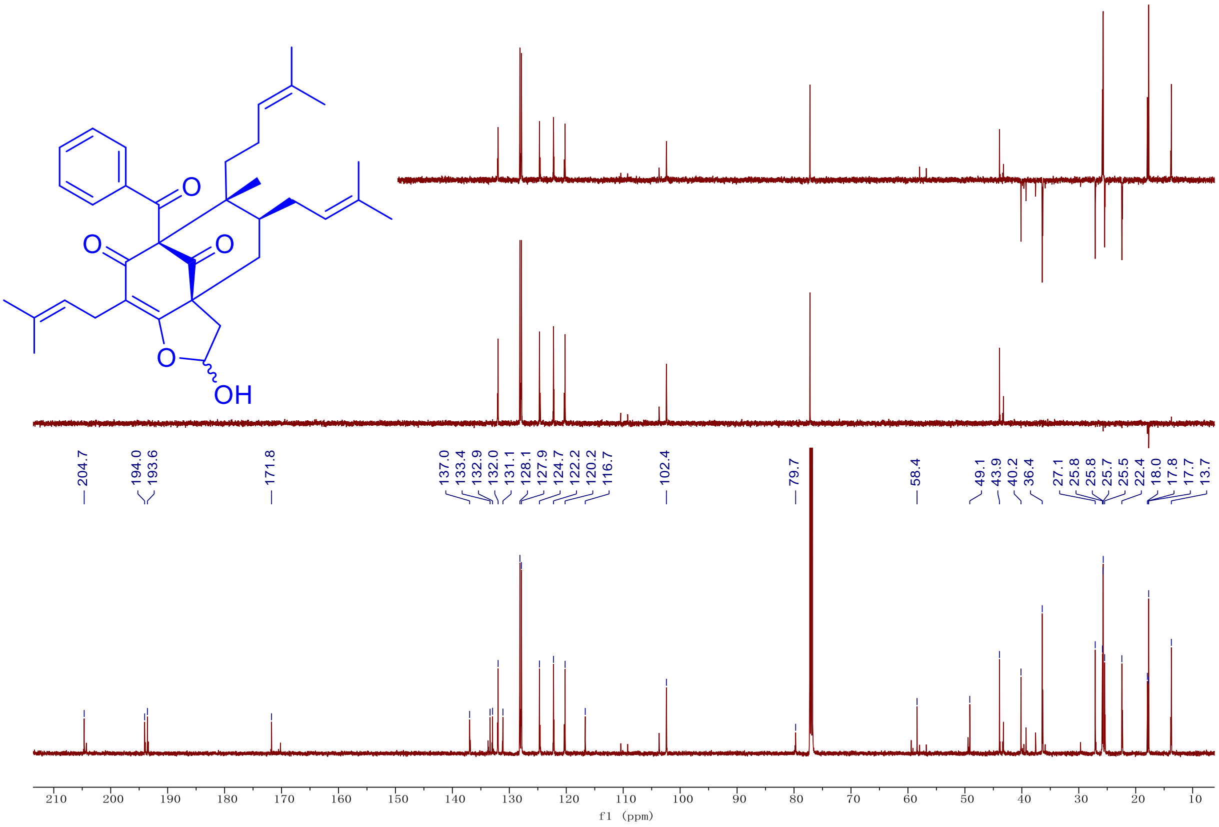

Figure S40. ^{13}C and DEPT NMR spectrum of **4** (in $CDCl_3$, 150 MHz).

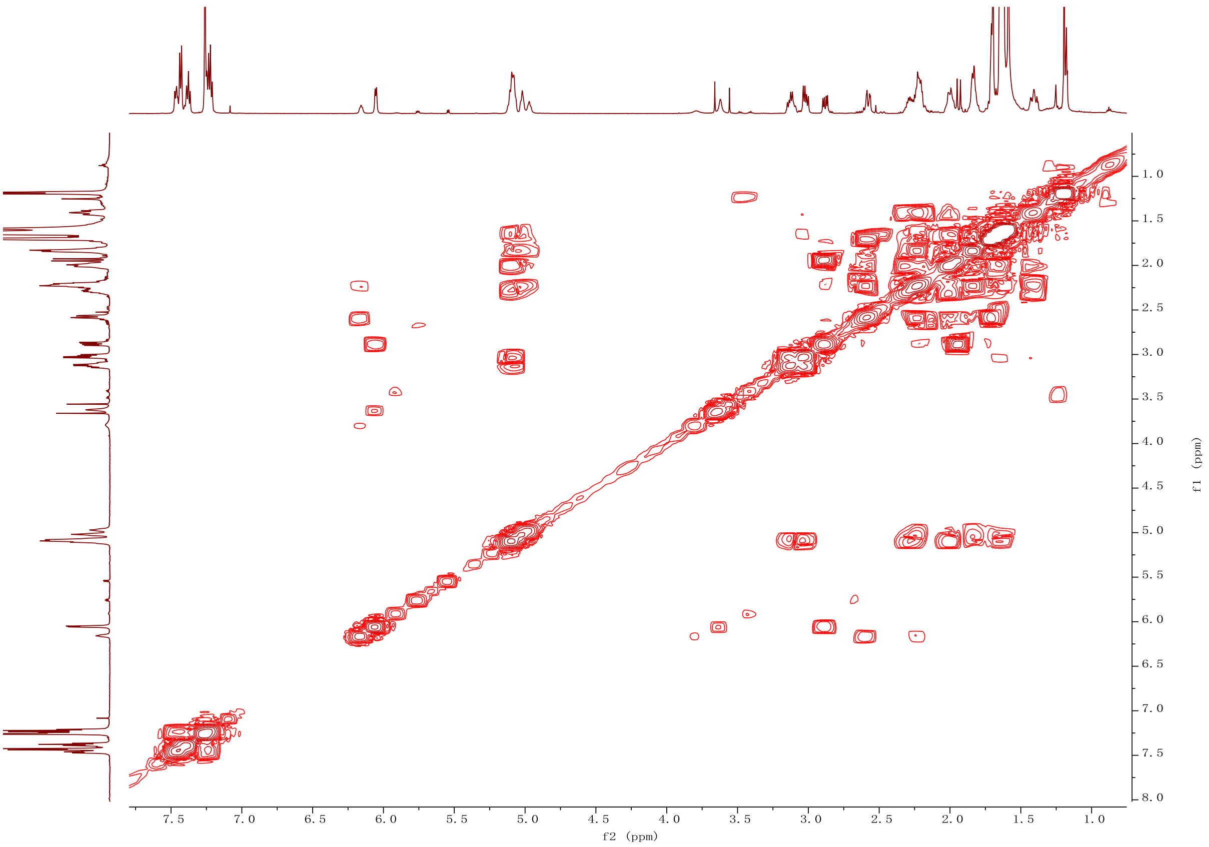

Figure S41. ^1H–^1H COSY spectrum of **4** (in $CDCl_3$, 600 MHz).

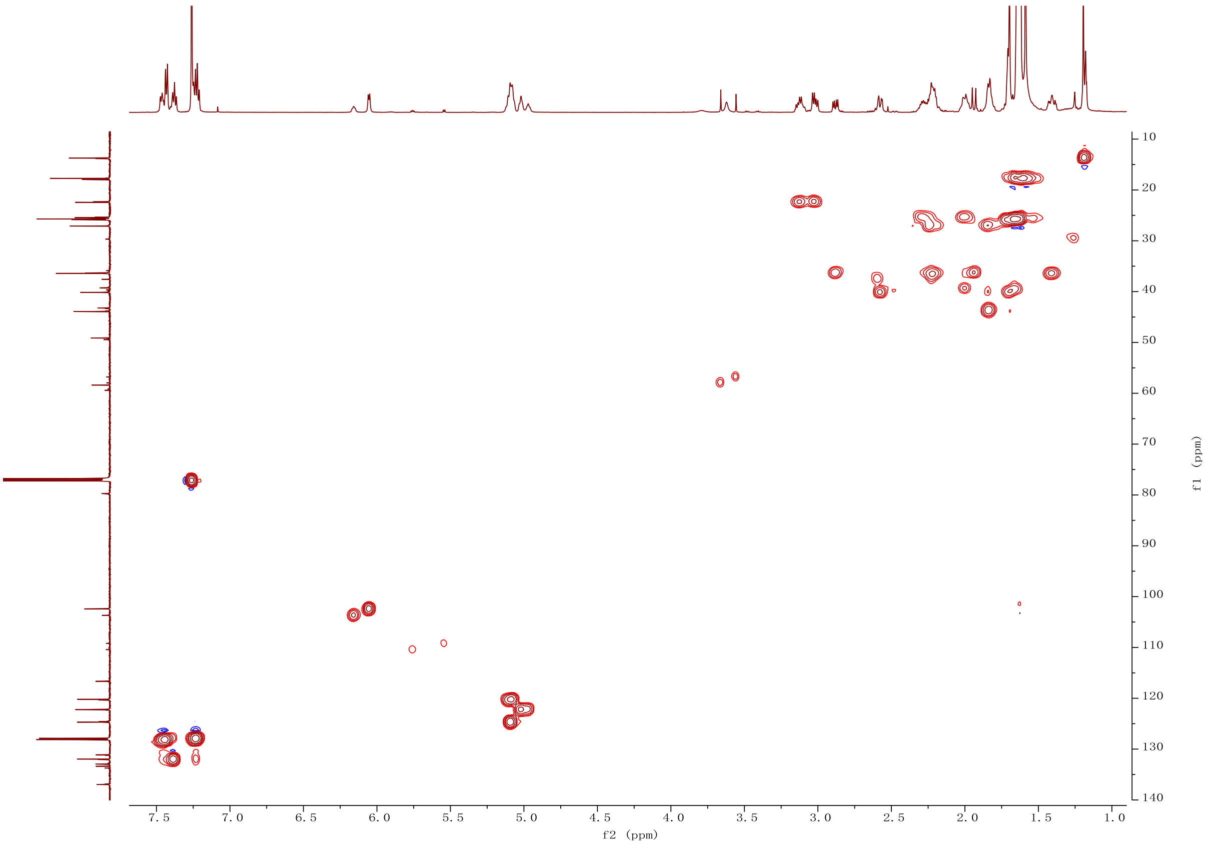

Figure S42. HSQC spectrum of **4** (in $CDCl_3$, 600 MHz).

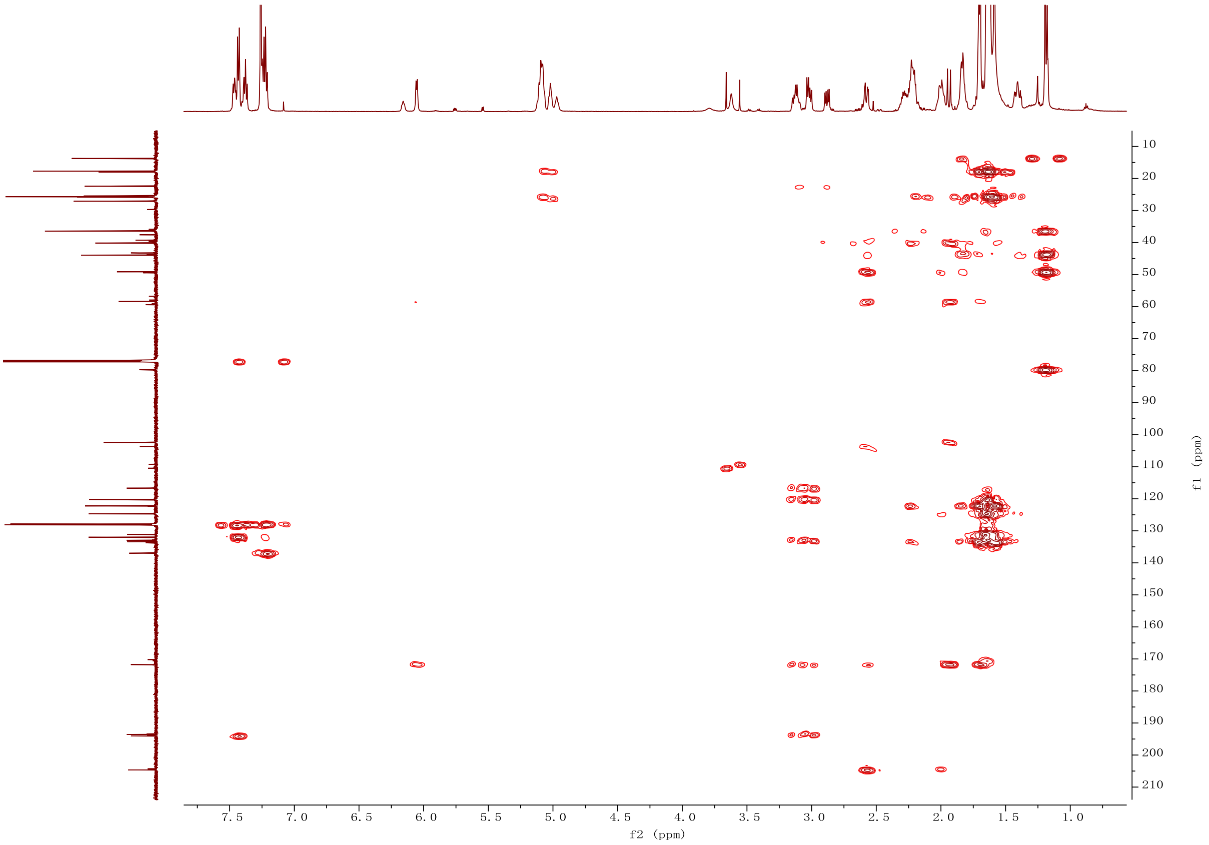

Figure S43. HMBC spectrum of **4** (in $CDCl_3$, 600 MHz).

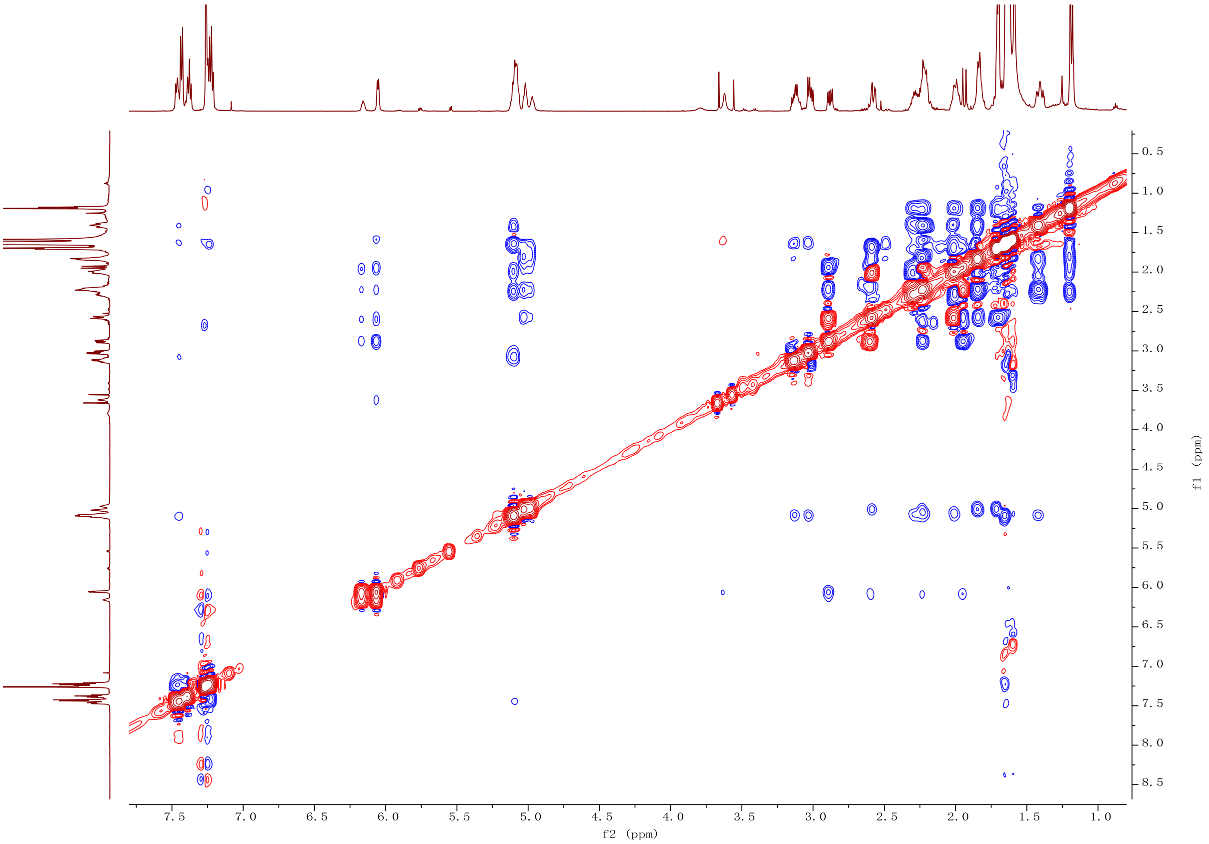

Figure S44. NOESY spectrum of **4** (in $CDCl_3$, 600 MHz).
